# BRD1 deficiency affects SREBF1-related lipid metabolism through regulating H3K9ac/H3K9me3 transition to inhibit HCC progression

**DOI:** 10.1038/s41419-025-07404-7

**Published:** 2025-02-17

**Authors:** Mingyang Zhang, Jing Bai, Hengye Yuan, Xiaojun Duan, Lei Yu, Yu Li, Kexin Li, Saqi Rile, Xinran Wang, Haisheng Wang, Pengxia Liu, Jia Yan, Changshan Wang

**Affiliations:** 1 College of Life Science, Inner Mongolia University, Xi Lin Guo Le south Road 49, Yu Quan District, Hohhot, Inner Mongolia China; 2 School of Basic Medicine, Inner Mongolia Medical University, Xin hua Street No. 5, Hui min District, Hohhot, Inner Mongolia China; 3 First School of Clinical Medicine, Inner Mongolia Medical University, Xin hua Street No. 5, Hui min District, Hohhot, Inner Mongolia China; 4 Medical Experimental Center of Basic Medical School, Inner Mongolia Medical University, Xin hua Street No. 5, Hui min District, Hohhot, Inner Mongolia China

**Keywords:** Cancer metabolism, Molecular biology

## Abstract

BRD1 encodes a protein containing a bromodomain, which is an essential component of histone acetyltransferase (HAT) complexes. These complexes play a crucial role in the regulation of gene transcription and the modification of chromatin structures. The aberrant expression of BRD1 is frequently observed across a range of cancer types, including hepatocellular carcinomas (HCC). However, the precise mechanisms through which BRD1 contributes to tumorigenesis, especially in HCC, remain unclear. In our investigation, we have uncovered a novel role for BRD1 as an oncogene implicated the regulation of lipid metabolism in HCC progression. Specifically, the deficiency of BRD1 impedes the proliferation and metastasis of HCC cells reducing the accumulation of lipid droplets and cholesterol levels. This effect is mediated through the SREBF1-induced downregulation of SCD1 expression in HCC cells. Mechanistically, the ablation of BRD1 disrupts acetylation level of H3K9, culminating in the subsequent trimethylation of H3K9 (H3K9me3). Notably, the H3K14ac partially colocalizes with H3K9me3 and its methyltransferase SETDB1 to from a double labeling of both H3K14ac and H3K9me3 at the SREBF1 promoter. This double labeling contributes to the creation of a repressive environment, ultimately leading to the downregulation of SREBF1 gene expression in HCC. Furthermore, the combinatorial use of a BRD1 inhibitor and simvastatin augments antitumor efficacy in vivo. Collectively, our findings underscore BRD1 as a critical regulator of SREBF1-associated lipid metabolism and a participant in HCC progression through a distinct epigenetic regulatory mechanism. These discoveries further suggest a promising epigenetic therapeutic approach for the treatment of HCC.

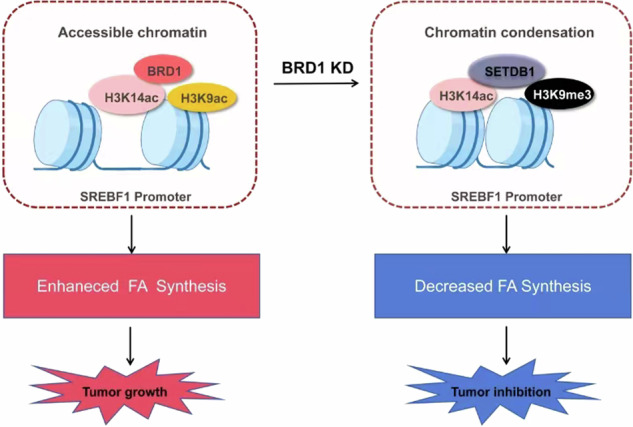

## Introduction

HCC is an aggressive human cancer and ranks among the most prevalent liver diseases, primarily resulting from chronic liver diseases [1]. Recent evidence increasingly indicates that metabolic-dysfunction-associated steatotic liver disease plays a significant role in the etiology of in HCC development [2]. It has been established that the initiation and progression of HCC are driven by metabolic reprogramming, with defects in hepatic lipid metabolism potentially leading to abnormal gene expression and triggering various cellular pathways involved in tumorigenesis and metastasis [3, 4]. Consequently, elucidating the metabolic regulatory mechanisms associated with HCC progression may facilitate the identification of novel diagnostic markers and therapeutic targets for patients with HCC.

HCC is widely recognized as a heterogeneous disease characterized by extensive epigenetic dysregulation, including chromatin remodeling, histone alterations, and DNA methylation. These epigenetic alterations are intricately linked to the progression and metastasis of HCC [5]. Various methyltransferases and demethylases, including EZH2, EHMT2, SETDB1 and SETD2, have been implicated in the clinical characteristics of HCC patients [6, 7]. Notably, the methylation of histone H3 at lysine 9 (H3K9) is frequently associated with the pathogenesis of HCC [8–10]. Furthermore, epigenetic mechanisms play pivotal roles in lipid metabolism, oxidative stress and inflammation through transcription dysregulation of lipid-related genes during HCC development. A global reduction in gene-specific histone H4 methylation at lysine 20 (H4K20me3) and acetylation at lysine 16 (H4K16ac) has been observed in the progression of nonalcoholic steatohepatitis (NASH)-related HCC [11]. HDAC8 interacts with the chromatin modifier EZH2 to synergistically inhibit Wnt antagonists via histone H4 deacetylation and H3 lysine 27 trimethylation, thereby activating transcription of lipogenic genes [12, 13]. Additionally, inhibition of HDAC3 decreases the acetylation of fatty acid synthase (FASN), leading to the suppression of HCC growth [14]. Thus, histone modification plays a critical role in the progression of liver cancer by modulating the expression of lipid metabolism-related genes.

The bromodomain containing 1 gene, BRD1 encodes an epigenetic regulator that plays a pivotal role in chromatin recruitment to modulate histone acetylation and, consequently, gene transcription. As a scaffold protein, BRD1 interacts with a range of epigenetic modifiers, including histone acetyltransferases KAT5 and KAT7, as well as the histone-lysine N-methyltransferase KMT5B [15, 16]. Notably, BRD1 forms a novel HAT complex with HBO1, responsible for the global acetylation of H3K14ac, which is essential for fetal liver erythropoiesis [17]. Additionally, BRD1 functions as a co-repressor of peroxisome proliferator-activated receptor (PPAR)-mediated transcription and its inactivation can lead to metabolic dysfunction [18]. Furthermore, downregulation or inhibition of BRD1 may enhance anti-tumor immune function by negatively regulating the activation states of T cells and natural killer (NK) cells. BRD1-mediated histone acetylation is also crucial for the activation of the CD8 gene during early thymocyte development [19]. Despite these advancements, the precise function of BRD1 that bridges its molecular mechanisms with tumor-related pathologies remains largely unexplored.

Sterol regulatory element-binding transcription factor 1 (SREBF1) is a pivotal transcription factor governing lipid synthesis, uptake, storage, and release, thereby maintaining lipid homeostasis and supporting rapid tumor growth [20]. SREBF1 directly activates the transcription of the FASN gene, leading to the accumulation of lipid droplets and promoting the development of HCC cells [21]. Previous research has primarily focused on SREBF1’s role as transcription factors controlling the expression of downstream genes, with evidence suggesting that it regulates hundreds of cis-regulatory elements across the squamous cancer epigenome, converging to activate cancer-promoting pathways [22]. Additionally, the SREBF1-CRTC2 complex potentially modulates the transcription of multiple proteins that fine-tune cellular metabolism [23]. Intriguingly, SREBF1 has been report to interact with the epigenetic marks in cancer development, recruiting KAT2A/GCN5 to deposit the epigenetic mark H2A-K130ac on SREBF1, thereby reigniting lipogenesis and steroidogenesis in prostate cancer [24]. However, the comprehensive epigenetic regulatory mechanism of SREBF1 remains elusive.

In this study, we demonstrate that BRD1 is involved in the epigenomic regulation of HCC. Through metabolomic and transcriptomic analyses, we show that BRD1 promotes lipid accumulation and tumorigenesis in HCC cells. Furthermore, we elucidate that SREBF1 serves as a central mediator linking BRD1 with fatty-acid metabolism. Mechanistically, BRD1 regulates SREBF1 expression in an H3K9ac dependent manner, but not H3K14ac dependent. Blocking BRD1 inhibits SREBF1 gene transcription by maintaining inhibitory chromatin through the formation of an H3K14ac-H3K9me3 co-modification, which is associated with a repressive chromatin state of SREBF1 gene, thereby inhibiting lipid synthesis and HCC growth. Collectively, our findings identify an epigenetic mode of action for BRD1 with H3K14ac-H3K9me3 in lipid metabolism, revealing BRD1 as a potential therapeutic target to affect lipid metabolism in HCC.

## Results

### BRD1 is markedly elevated and functions as an oncogenic factor in the progression of HCC

**Fig. 1:**
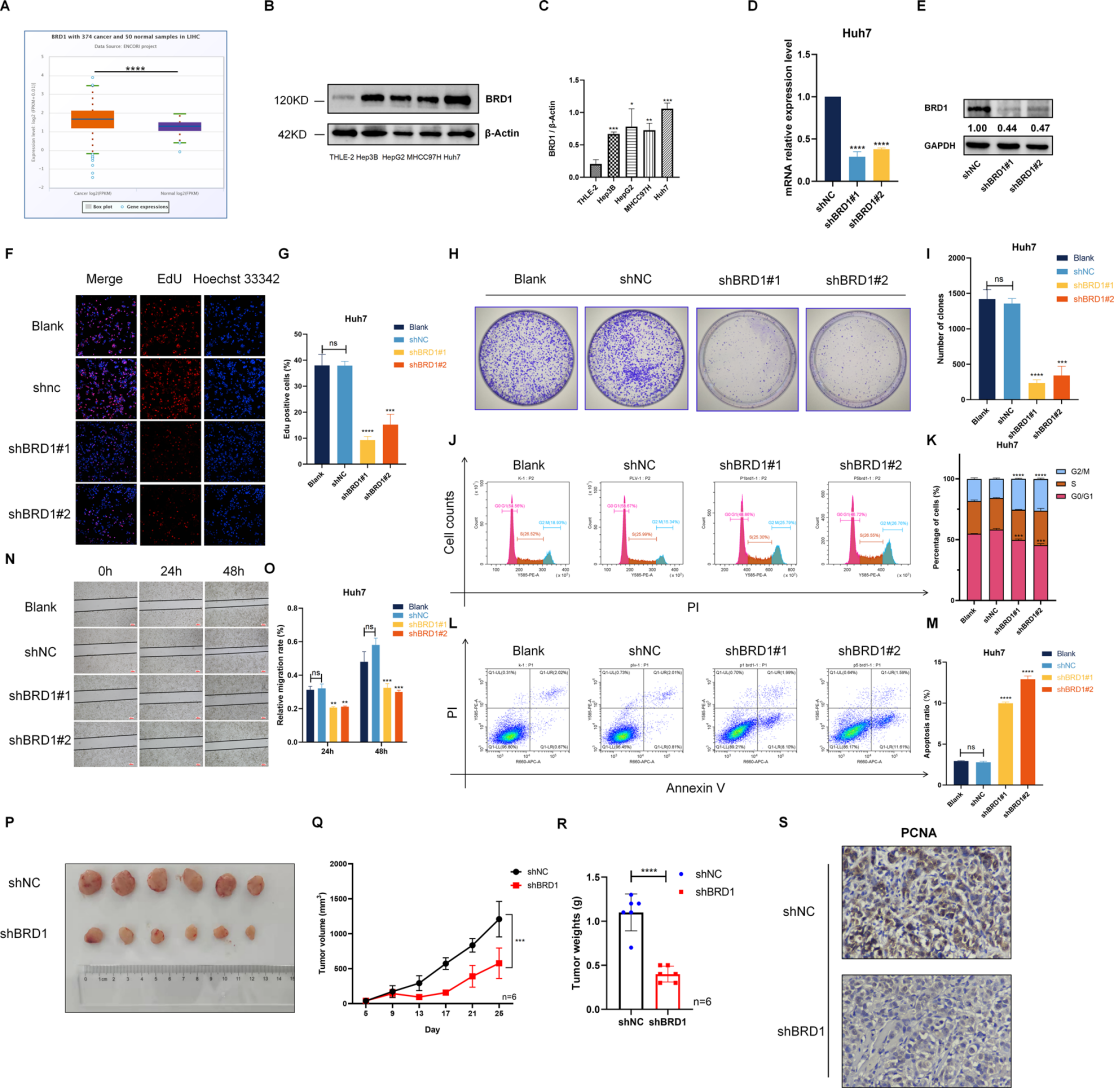
BRD1 promotes HCC cell growth and migration in vitro and in vivo. **A** BRD1 expression was elevated in LIHC compared to normal samples. **B**, **C** The expression levels of BRD1 were assessed in various HCC cell lines. **B** protein level, **C** mRNA level. **D**, **E** Detection of BRD1 gene knockout efficiency, **D** mRNA level, **E** protein level. **F**, **G** Cell viability rates were compared after BRD1 downregulation versus control conditions. **F** The detection of Edu-labeled DNA was performed using a con-focal microscope. **G** Cell viability rates statistics. **H**, **I** The colony-forming ability of Huh7 cells was analyzed using a clone formation assay. **J**, **K** The cell cycle distribution was determined following BRD1 downregulation. Representative images from cell cycle assays in the indicated cell lines are shown, along with statistical analysis in the adjacent panel. **L**, **M** Cell apoptosis was assessed by analyzing Annexin V-FITC and PI staining. **N**, **O** HCC cells transfected with BRD1 shRNA exhibited reduced motility in a wound healing assay compared to control cells. **P**–**R** Downregulation of BRD1 inhibited the growth of MHCC97-H cell xenografts in nude mice. **P** Representative tumors, **Q** tumor volumes, and **R** tumor weights in each group. **S** IHC staining of *PCNA* gene expression in tumor tissues excised from nude mice. The number of mice per group was six (*n* = 6). Data are presented as the mean ± standard deviation (SD) of six independent measurements. Images from each group are depicted at an original magnification of 200×; scale bars represent 100 micrometers. Data are presented as the mean ± SD of three independent experiments. Statistical significance is denoted as ns *p* > 0.05, *p < 0.05,  **p < 0.01, ***p < 0.001, and ****p < 0.0001.

To elucidate the function role of BRD1 in the tumorigenesis of HCC, we first confirmed the expression status of BRD1 in liver hepatocellular carcinoma (LIHC) tissue. We found that the mRNA level of BRD1 was significantly upregulated in HCC tissues based on the patient’s data with HCC in TCGA database (Fig. 1A). Moreover, BRD1 was significantly increased in patients with a more advanced tumor grade, especially grade 3, suggesting that BRD1 expression was significantly correlated with high-grade 3 tumor (Figure S1). Furthermore, we detected protein level of BRD1 in various HCC cell lines including Hep3B, HepG2, MHCC97H, and Huh7. BRD1 was also elevated in HCC cell lines, including Hep3B, HepG2, MHCC97H, and Huh7, compared with the normal liver cells (Fig. 1B, C). Collectively, these results suggest that the abnormal expression of BRD1 likely play an important role in liver tumor progression.

To explore the potential oncogenic role of BRD1 in HCC development, we generated BRD1-downregulated cells by employing two BRD1-specific short hairpin RNAs (shBRD1#1 and shBRD1#2) in HCC cell lines (Fig. 1D, E). The results of Edu staining and colony formation assays reveal that the cell proliferation ability was significantly decreased in BRD1 downregulated HCC cells (Fig. 1F–I). Furthermore, cell cycle analysis demonstrated a significant G2-M phase arrest in cells following BRD1 inhibition (Fig. 1J, K). Notably, an increase in early-stage cell apoptosis was observed in BRD1-downregulated HCC cells (Fig. 1L, M). Additionally, the wound healing assays indicated a notable impairment in the migration ability of HCC cells upon downregulation BRD1 deficiency (Fig. 1N, O). Furthermore, we demonstrated that knocking down BRD1 in Hep3B cells significantly inhibited cell proliferation and metastatic capacity, while promoting apoptosis (Figure S2a–j). These results collectively suggest that BRD1 downregulation significantly reduces cell proliferation and migration while promoting cell apoptosis in HCC.

To further validate the oncogenic effect of BRD1 in vivo, we conducted experiments that demonstrated a significant reduction in tumor volumes and weights upon BRD1 knockdown, indicating effective tumor regression (Fig. 1P–R). Moreover, the result of immunohistochemical (IHC) staining also revealed a marked decrease in PCNA protein levels in tumor tissue following BRD1 inhibition (Fig. 1S). In conclusion, BRD1 is significantly upregulated in HCC and functions as an oncogenic factor that contributes to the malignant progression of this disease.

### Knockdown of BRD1 influences lipid metabolism and disrupts cholesterol homeostasis in HCC

**Fig. 2:**
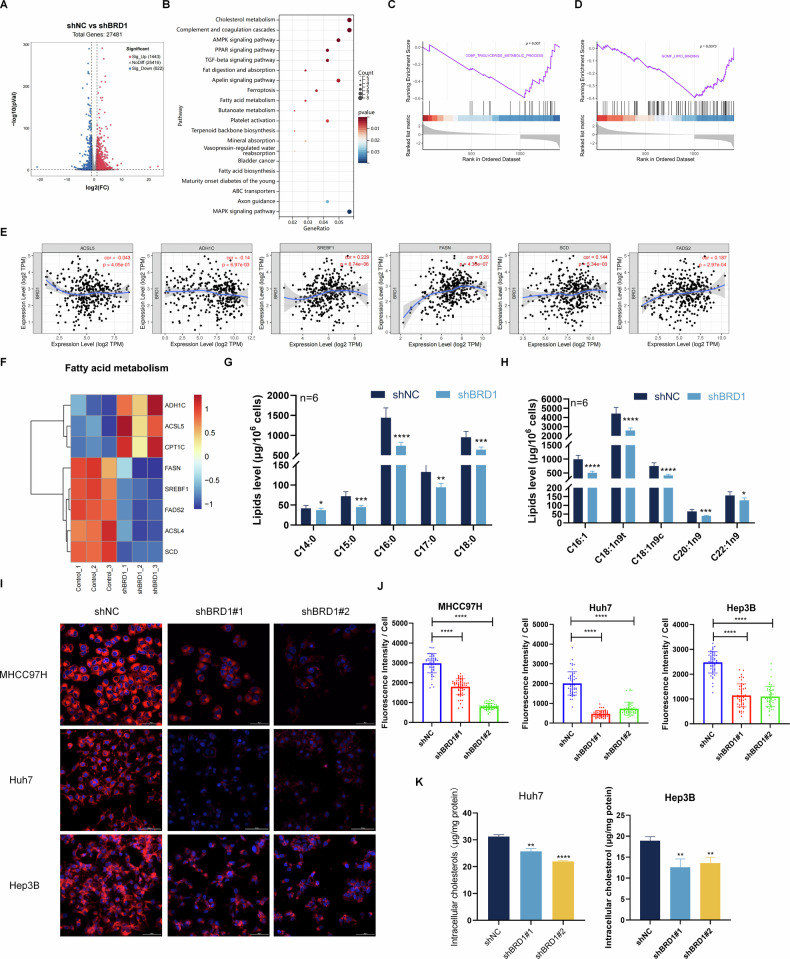
BRD1 plays a pivotal role in fatty-acid and cholesterol metabolism. **A** RNA-sequencing and bioinformatics analysis were conducted on BRD1-downregulated HCC cells. DEGs were identified. **B** KEGG enrichment analysis was performed on the representative pathways of the DEGs identified. **C**, **D** GSEA enrichment analysis revealed that BRD1-related DEGs are implicated in metabolic-related pathways, with *p*-value < 0.05 indicating statistical significance. **E** An analysis was conducted to investigate the correlation between BRD1 expression and genes related to fatty-acid and cholesterol metabolism in LIHC. **F** A heat map was generated to illustrate the gene expression level of differential genes associated with fatty acid metabolism. **G**, **H** Metabolic alterations were observed in BRD1-downregulated HCC cells, specifically changes in the proportions of saturated and monounsaturated fatty acids were detected (**G**), and the proportion of monounsaturated fatty acids was analyzed (**H**). **I**, **J** Nile red staining was used to visualize the accumulation of lipid droplets in HCC cells after BRD1 inhibition. **K** The proportion of total intracellular cholesterol assessed following the inhibition of BRD1. All data presented are the mean ± SD from three independent experiments. *p < 0.05; **p < 0.01; ***p < 0.001; ****p < 0.0001.

To elucidate the role of BRD1 in the progression of HCC by examining gene expression profiles, BRD1 knockdown HCC cells was generated and subjected them to RNA sequencing (RNA-seq) analysis. Our RNA-seq data analysis revealed 1443 upregulated genes and 622 downregulated genes following BRD1 inhibition in HCC cell (Fig. 2A). To gain insights into the functional implications of these differentially expressed genes (DEGs), we conducted Kyoto Encyclopedia of Genes and Genomes (KEGG) enrichment analysis. The results indicated that these genes were implicated in cholesterol metabolism, fat digestion and absorption, and fatty acid (FA) metabolism (Fig. 2B). Notably, fatty acid metabolism-related pathways, include PPAR, AMPK, MAPK, and TGF-beta signaling pathways, were observed in the BRD1-related RNA-seq results (Fig. 2B). Gene Set Enrichment Analysis (GSEA) results indicated that these genes were significantly associated with the triglyceride metabolic processes and lipid-binding pathways (Fig. 2C, D). Furthermore, we found that the expression of fatty acid metabolism-related genes, *ADH1C* and *ACSL5*, were negatively correlated, while *FASN*, *SREBF1*, *FADS2*, *ACSL4*, and *SCD1* were positively correlated with BRD1 expression in HCC (Fig. 2E). Consistent with these finding, the mRNA level of *ADH1C* and *ACSL5* was increased, whereas those of *FASN*, *SREBF1*, *FADS2*, *ACSL4* and *SCD1* were decreased in BRD1-downreguted HCC cells (Fig. 2F). These data collectively suggest that BRD1 is likely involved in the regulation of fatty acid and cholesterol metabolism in HCC.

To further investigate the involvement of BRD1 in lipid metabolism, we performed gas chromatography-mass spectrometry (GC-MS). A metabolomic comparison between negative control (NC) and those BRD1-knockdown cells was perform in HCC. Our fundings reveals a significant decrease in the content of various free fatty acid in BRD1-knockdown cells. Specifically, the levels of saturated fatty acids such as pentadecanoic acid (C15:0) and palmitic acid (C16:0), as well as monounsaturated fatty acids including palmitoleic acid (C16:1) and oleic acid (C18:1), were markedly reduced BRD1-knockdown cells (Fig. 2G, H).

Furthermore, the lipid droplets were observed using confocal fluorescence in HCC cells with downregulated BRD1 expression HCC cells. Nile red staining demonstrated that inhibiting BRD1 effectively suppressed the accumulation of lipid droplets in these cells (Fig. 2I, J). Moreover, our analysis indicated a significant reduction in total cellular cholesterol levels of BRD1-silenced HCC cells compared to control cells (Fig. 2K). Taken together, our multi-omics and biochemical results confirmed the critical roles of BRD1 in lipid accumulation and cholesterol homeostasis regulation in HCC cells.

### BRD1 deficiency attenuates lipid metabolism and cholesterol homeostasis via regulating SREBF1-FASN/SCD1 axis

**Fig. 3:**
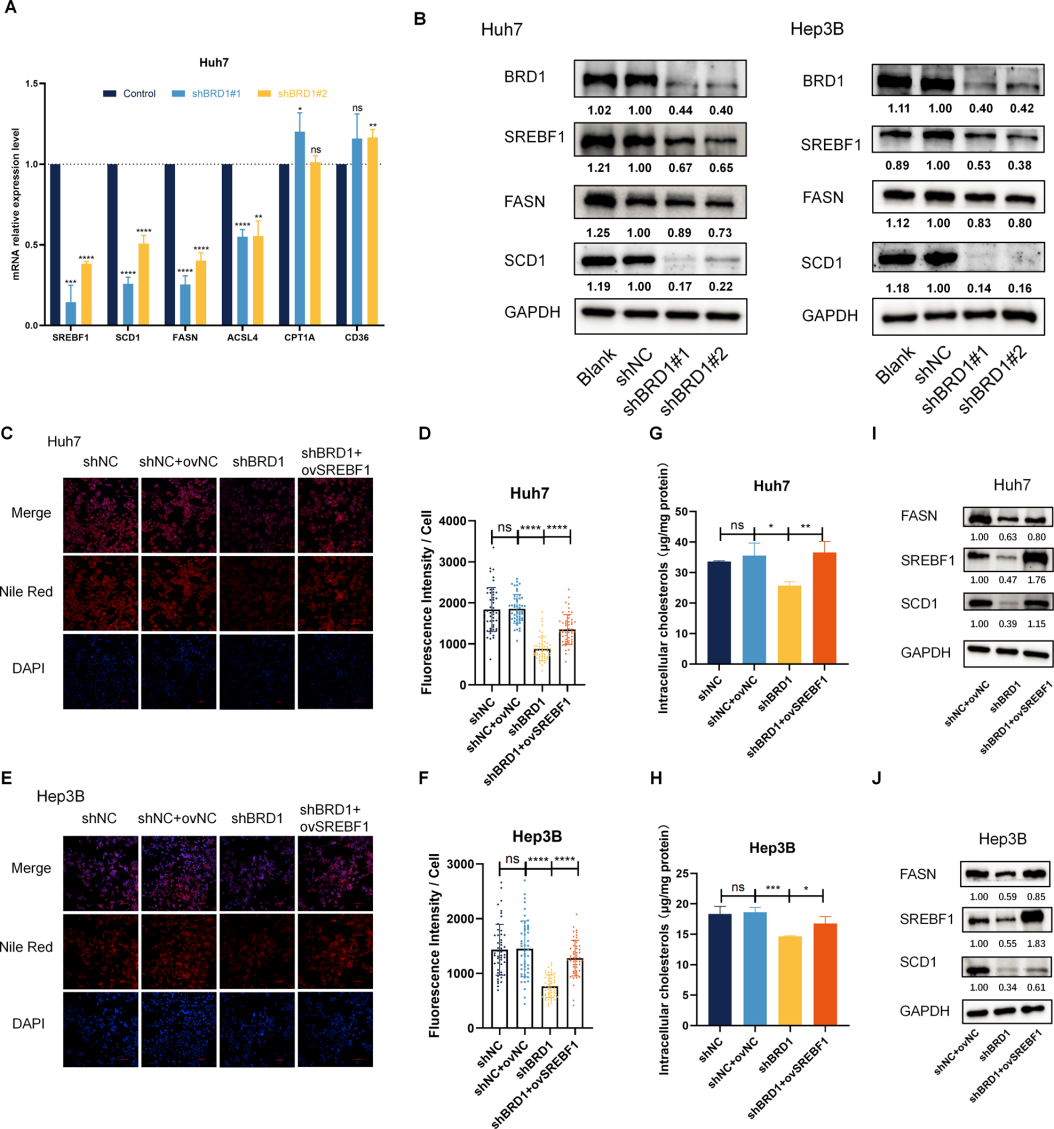
BRD1 regulates fatty-acid and cholesterol metabolism pathways through SREBF1 in HCC. **A**, **B** Expression level of *SREBF1*, *FASN*, *SCD1*, *ACSL4*, *CPT1A* and *CD36* were analyzed in HCC cells downregulated BRD1. **A** mRNA level in Huh7 cell, **B** protein level in Huh7 and Hep3B cells. **C**–**F** Nile red staining to detect the accumulation of lipid droplets after inhibition of BRD1 or overexpression of SREBF1 while inhibiting BRD1 in HCC cells. **C**, **D** in Huh7 cell, **E**, **F** in Hep3B cell. **G**, **H** The total cholesterol level was measured in HCC cells transfected with control vector, shBRD1 vector, or shBRD1 vector combined with SREBF1-overexpressing vector. **G** in Huh7 cell, and **H** in Hep3B cell. **I**, **J** Western blot shows the protein levels of SREBF1, FASN, and SCD1 in HCC cells transfected with control vector, shBRD1 vector, or shBRD1 vector in combined with SREBF1-overexpressing vector. **i** in Huh7 cell, and **J** in Hep3B cell. All data presented are the mean ± SD from three independent experiments. ns *p* > 0.05; *p < 0.05; **p < 0.01; ***p < 0.001; ****p < 0.0001.

To elucidate the mechanisms by which BRD1 influences lipid biosynthesis and cholesterol homeostasis, we conducted a comprehensive analysis of differentially expressed genes related to BRD1 in HCC cells. We found that the mRNA and protein levels of SREBF1, SCD1, FASN, were significantly downregulated after BRD1 knockdown in Huh7 cell (Fig. 3A, B). Previous study has established SREBF1 as a master regulator of cholesterol and lipid biosynthesis-related gene expression [25]. To verify whether this pattern is also present in BRD1-deficient HCC, we overexpressed SREBF1 in BRD1-downregualted HCC cell. Nile red staining results showed that BRD1 knockdown inhibited intracellular lipid accumulation, an effect that was partially rescued by SREBF1 overexpression in HCC cell (Fig. 3C–F). Additionally, overexpression of SREBF1 partially restored the cholesterol content of BRD1 knockdown HCC cells (Fig. 3G, H). Collectively, these findings implicate BRD1 in the regulation of lipid metabolism and cholesterol homeostasis through modulation of SREBF1 in HCC.

Previous studies have highlighted that the pivotal role of SREBF1 in regulating FA synthesis by transactivating enzymes involved in this process, functioning as a transcription factor to control the expression of target genes such as FASN, SCD1, and ACC [26]. To investigate whether BRD1 regulates the lipogenic enzyme SCD1 and FASN via SREBF1, we assessed the expression of these enzymes in cells with SREBF1 over-expression and BRD1 knockdown. The qPCR results indicated that the mRNA level of *FASN* and *SCD1* genes was increased following the overexpression of SREBF1 in BRD1 knockdown HCC cells (Figure S3). Moreover, the decrease in FASN and SCD1 protein levels resulting from BRD1 knockdown was restored by the overexpression of SREBF1 in HCC cells (Fig. 3I, J). These findings indicate a positive correlation between BRD1 and SREBF1-mediated lipid accumulation, implicating BRD1 in the regulation of SCD1 and FASN in HCC.

### The BRD1-SREBF1 mediated lipid metabolic reprogramming drives malignant progression in HCC

**Fig. 4:**
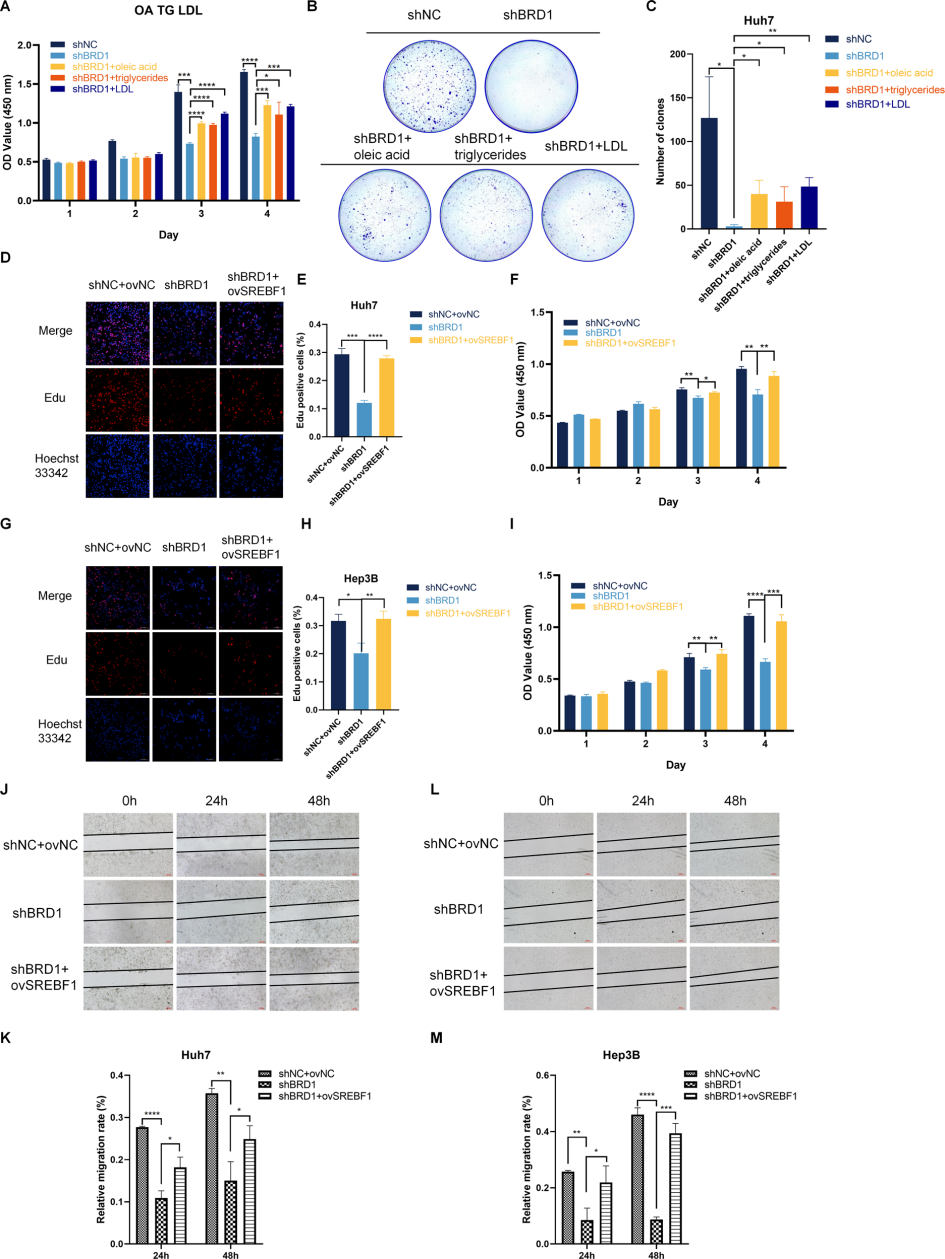
BRD1 promotes HCC progression via SREBF1-related metabolism pathways. **A**–**C** The proliferation of BRD1-downregulated Huh7 cells, treated with oleic acid, triglyceride and low-density lipoprotein. A CCK-8 assay was used to quantify cell viability (**A**). B, C colony-formation assays to assess clonogenic growth potential. **D**–**I** The proliferation of Huh7 cell and Hep3B cell, transfected with SREBF1 overexpression vector while BRD1 was downregulated, was evaluated. **D**–**H** EdU assays to visualize DNA synthesis and cell proliferation. **D**, **E** in Huh7 cell,and **G**, **H** in Hep3B cell. **F**–**I** CCK-8 assay to quantify cell viability under these conditions in HCC cell, **F** in Huh7 cell, and I in Hep3B cell. **J**–**M** HCC cells transfected with SREBF1 overexpression vector while BRD1 was downregulated, was evaluated cell migration ability using a wound healing assay compared to control cells. **G**, **K** in Huh7 cell, **L**, **M** in Hep3B cell. Data are presented as the mean ± SD from three independent experiments. *p < 0.05; **p < 0.01; ***p < 0.001; ****p < 0.0001.

We further validated the involvement of BRD1 in metabolic regulation and its role in facilitating the malignant progression of HCC. In BRD1-knockdown cells, the supplementation of exogenous oleic acid, triglycerides, and low-density lipoproteins reversed the inhibitory effects of BRD1 knockdown on cell proliferation and colony formation in HCC cells (Fig. 4A–C). These observations underscore the role of BRD1 in promoting HCC tumorigenesis through the regulation of lipid metabolism.

To investigate whether the carcinogenic effect of BRD1 dependent on SREBF1, we enhanced SREBF1 expression in BRD1-silenced cells. CCK8 and EdU assays indicated that the overexpression of SREBF1 significantly rescued the inhibitory effects of BRD1 knockdown on HCC cell proliferation (Fig. 4D–I). Moreover, the wound healing assay demonstrated that BRD1 knockdown suppressed the cell migration, an effect that was reversed by SREBF1 overexpression in HCC cells (Fig. 4J–M). These results indicate that BRD1 regulates SREBF1 expression, thereby modulating lipogenesis to promote cell proliferation and migration in HCC cells.

### BRD1 deficiency inhibits SREBF1 expression by influencing the SETDB1-dependent transition between H3K9ac and H3K9me3 in HCC

**Fig. 5:**
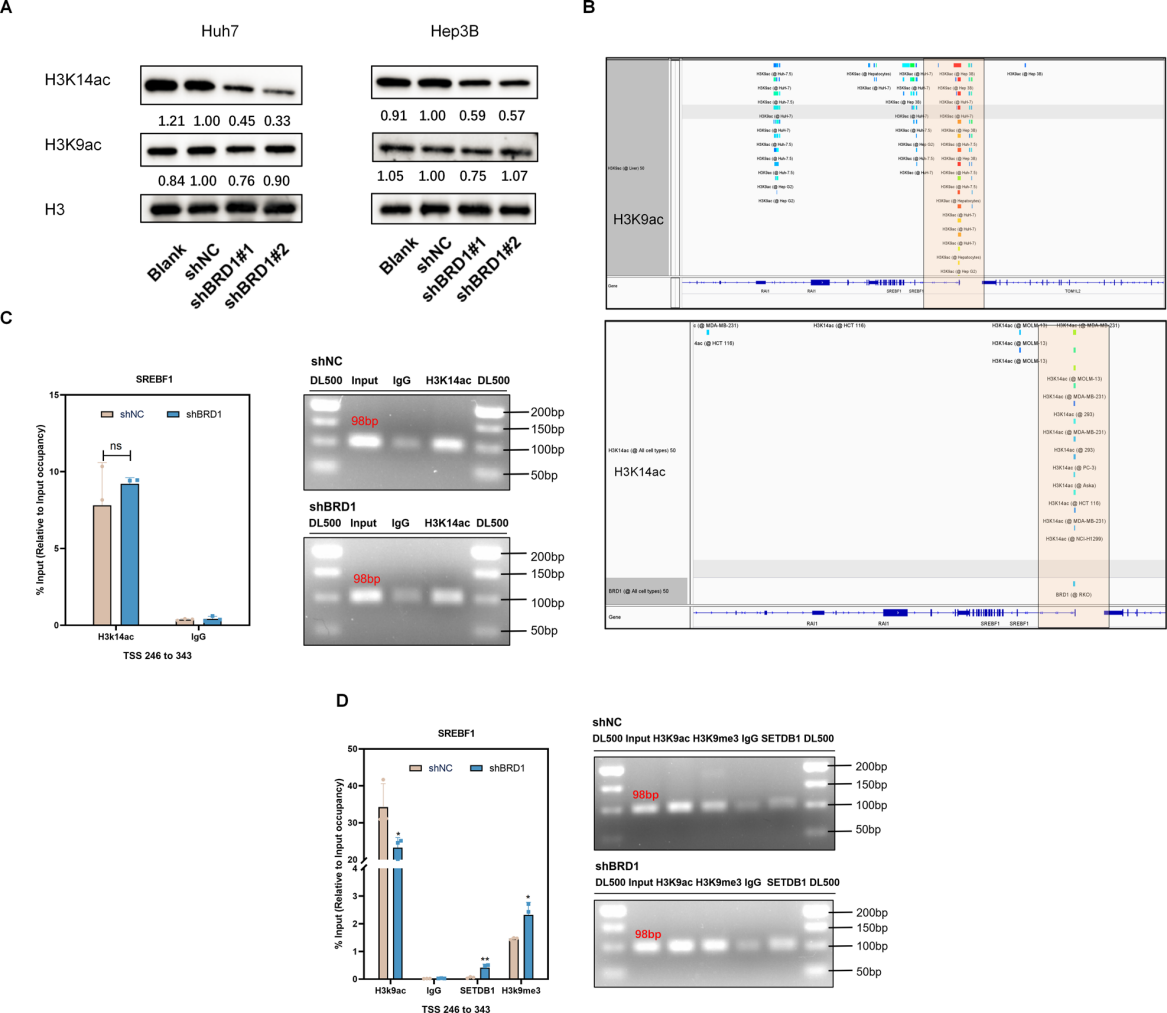
BRD1-related SREBF1 expression regulation is dependent on epigenetic regulation of H3K9 and H3K14 loci. **A** Immunoblotting was performed to assess the total protein levels of H3K14ac and H3K9ac in Huh7 and Hep3B cells. **B** The integrative genomics viewer (IGV) was used to visualize the genomic enrichment of H3K14ac and BRD1 across all cells, based on data retrieved from the ChIP-Atlas database. Shaded areas Shaded areas highlight the enrichment of H3K9ac and H3K14ac specifically at the SREBF1 promoter region. **C**, **D** The enrichment of H3K14ac, H3K9ac, H3K9me3, and SETDB1 in the SREBF1 promoter region was detected by ChIP-qPCR. The figures on the right depict the band size of SREBF1, detected by nucleic acid electrophoresis using shNC and shBRD1 ChIP-DNA as templates. The product length observed is 98 base pairs (bp). Data represent the mean ± SD of three independent experiments. ns *p* > 0.05; *p < 0.05; **p < 0.01.

BRD-containing proteins play crucial roles in gene transcription by acetylating histone H3K9 and H3K14 at the gene promoters [15]. In our study, we detected the overall changes in H3K9ac and H3K14ac and found that the levels of H3K14ac significantly decreased, while there was no significant change in H3K9ac levels. (Fig. 5A). To elucidate the mechanism by which BRD1 regulates SREBF1 expression, we analyzed the occupancy of H3K9/14ac on the SREBF1 promoter. Bioinformatics analysis revealed an enrichment region of H3K9ac, H3K14ac and BRD1 at the SREBF1 promoter based on the human cell Chip-Atlas data (Fig. 5B). Therefore, we speculated that BRD1 likely influences the expression of SREBF1 by regulating the levels of H3K14ac or H3K9ac at the promoter. Subsequently, our ChIP-qPCR results demonstrated that downregulation of BRD1 did not significantly reduce H3K14ac levels, but did result in a decrease in H3K9ac level on SREBF1 promoter following a reduction of BRD1 enrichment at the SREBF1 promoter. (Fig. 5C, D and S4). These results suggest that BRD1 regulates SREBF1 expression through H3K9ac, but not H3K14ac level in HCC cells.

Interestingly, the knockdown of BRD1 does not affect the occupancy of H3K14ac but decreases the level of H3K9ac at the SREBF1 promoter. Previous study studies have reported that the co-occurrence of H3K9me3 and H3K14ac in histone H3 isolated from various human cell types [27–29]. Notably, regions marked by both H3K9me3 and H3K14ac are defined as poised inactive states [27]. We hypothesized that BRD1 deletion may be associated with the dual marking of H3K9me3 and H3K14ac in regulating SREBF1 expression in HCC. To further validate this hypothesis, we examined the occupancy of H3K9me3 at the BRD1 binding site of the SREBF1 promoter. Our results showed that the H3K9me3 signal level was significantly enhanced in BRD1 downregulated HCC cells (Fig. 5D).

Since SETDB1 has been confirmed to be associated with H3K9me3 and H3K14ac dually marked regions to result in the formation of a silenced chromatin environment [27], we detected the occupancy of SETDB1 in this region of the SREBF1 promoter. The ChIP-qPCR results demonstrated a significant enrichment of SETDB1 was observed in the region occupied by H3K9me3 at the SREBF1 promoter (Fig. 5D). Therefore, the repression of SREBF1 transcription induced by BRD1 inhibition is primarily attributed to the dual occupancy effect of H3K9me3 and H3K14ac at the SREBF1 promoter, meanwhile the conversion of H3K9ac to H3K9me3 is mediated by SETDB1.

### Synergistic inhibition of HCC cell growth by combination therapy with a BRD1 inhibitor and simvastatin

**Fig. 6:**
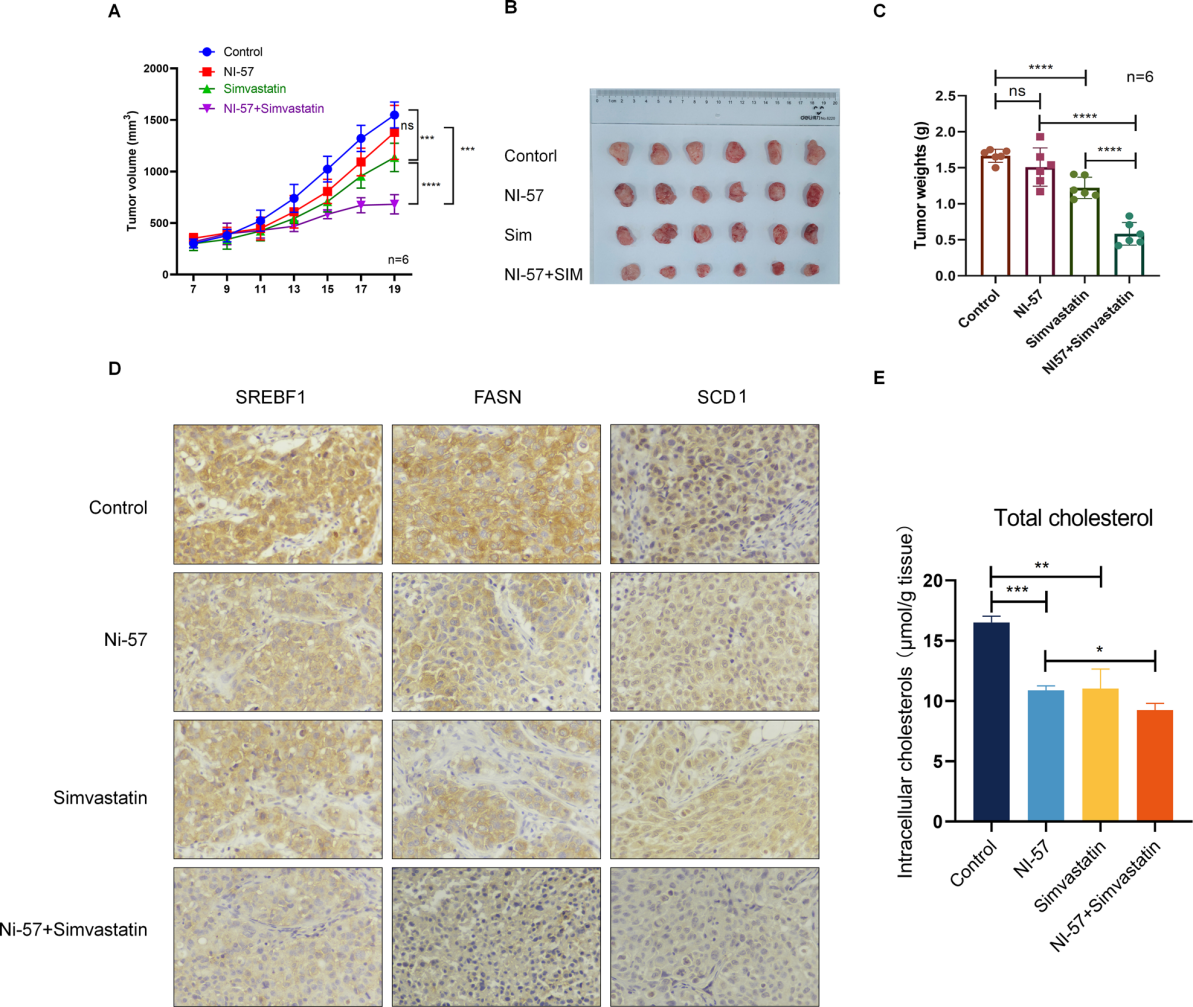
BRD1 inhibitor combined with Simvastatin synergically inhibit HCC progression. **A** The concurrent administration of BRD1 inhibitors and simvastatin exerts a significant suppressive effect on the tumor growth of HCC cell xenografts in nude mice. **A** BRD1 inhibitors and simvastatin inhibits tumor growth. **B** Tumor volume. **C** The tumor weight in each group was assessed, confirming the synergistic inhibitory action of BRD1 inhibitors and simvastatin, particularly when used in combination, on HCC tumor burden. **D** IHC staining of tumor tissues excised from nude mice demonstrates that the expression level of SREBF1, FASN, and SCD1 proteins were significantly downregulated following treatment with BRD1 inhibitors and simvastatin. Each experimental group consisted of 6 mice (*n* = 6), ensuring the robustness and reproducibility of the observed results. **E** The analysis of total cholesterol content in tumor tissues of xenograft tumor models (*n* = 3). The number of mice in figure a-d was 6 (*n* = 6). Data represent the mean ± SD of three independent experiments. ns *p* > 0.05; *p < 0.05; **p < 0.01; ***p < 0.001; ****p < 0.0001.

To further elucidate the function role of BRD1 in vivo, we constructed a mouse xenograft model of HCC and treated it with the BRD1 inhibitor (iBRD1, NI-57). Although the tumor volume did not exhibit a significant reduction in the NI-57-treated group (Fig. 6A–C), IHC results indicated a notable decrease in SREBF1 expression within the tumor tissues following iBRD1 treatment. Concurrently, the expression levels of downstream genes, FASN and SCD1, were also downregulated in the NI-57-treated groups (Fig. 6D). Moreover, the total cholesterol level of tumor tissue was decreased after iBRD1 treatment (Fig. 6E). These results suggest that the inhibition of BRD1 may represent an effective strategy for modulating SREBF1 expression in vivo.

Here, we investigated the potential of combination strategies to enhance the therapeutic efficacy of iBRD1 in HCC. Simvastatin (SIM), a medication known for its cholesterol-lowering properties, has been shown to inhibit cholesterol synthesis and exhibit anticancer effects in the treatment of HCC [30, 31]. We thus evaluated the impact of combining simvastatin with iBRD1 on HCC growth in vivo. Notably, the simvastatin and iBRD1 combination exerted the most potent tumor suppressive effects compared to the single-agent treatments (Fig. 6A–C). Furthermore, IHC results also demonstrated that the combined treatment significantly inhibited the expression of metabolic genes SREBF1, SCD1, and FASN in xenografted tumors (Fig. 6D). Additionally, we found that the total cholesterol content in tumor tissues also decreased due to the effects of NI-57 and simvastatin, and the combined use of the two drugs had a greater impact on cholesterol levels than the use of NI-57 alone (Fig. 6E). These results suggest that the combination of iBRD1 and SIM exhibits a synergistic inhibitory effect on HCC cell growth, indicating a potential therapeutic strategy for liver cancer treatment.

## Discussion

BRD-containing proteins constitute a pivotal class of epigenetic readers with crucial implications across various cancer types. Notably, among the BRD family, BRD4, BRD8 and BRD9 have been implicated as potential prognostic indicators and druggable epigenetic targets in HCC [32]. In our study, we found that BRD1 level was significantly elevated in HCC patients, especially in grade 3. However, this elevation was not observed in grade 4, nor did we find a meaningful correlation between aberrant BRD1 expression and the prognosis of HCC patients. Additionally, we found that BRD1 gene is scarcely expressed in normal liver cells. This upregulation of BRD1 in HCC presents an intriguing observation. These results suggest that BRD1-mediated epigenetic regulation has a dual role in different grades of HCC progression. We hypothesize that the effects of BRD1 on cancer progression might be modulated through interactions with other molecules, which can obscure its direct impact on patient outcomes. Thus, BRD1 likely functions as an integral component within a broader network of genes and proteins, each playing distinct roles at different stages of tumor progression.

In this study, we demonstrated that knocking down BRD1 expression led to a reduction in cell proliferation, metastasis, and tumor growth, both in vitro and in vivo. Additionally, BRD1 knockdown induced cell cycle arrest and apoptosis. Moreover, our study revealed that downregulation of BRD1 inhibits fatty acid accumulation and cholesterol homeostasis in HCC cells, thereby suppressing tumorigenesis. Notably, the influence of BRD1 on lipid metabolism in HCC has not been previously reported. These findings underscore the indispensable role of BRD1 in HCC metabolism, as its depletion appears to impair lipid synthesis and hinder cell growth and metastasis.

Over the past decade, the prevalence of HCC is also increased due to metabolic risk factors, such as obesity and NAFLD [4, 6]. Metabolic deregulation is now recognized as a defining characteristic of cancer, with alterations in lipid metabolism gaining increasing attention. Our study found that the expression of lipid metabolism-related genes, including SREBF1, FASN and SCD1, was decreased in BRD1 downregulated HCC cell. These genes encode lipogenic enzymes that are frequently expressed in various cancers including HCC, and are implicated in tumorigenesis. Importantly, targeting FASN and SCD1 has shown promise as a therapeutic strategy for HCC [33, 34]. Thus, our findings suggest that BRD1 may represent a promising therapeutic target for HCC by modulating lipid metabolism.

SREBF1, the pivotal upstream molecule of lipogenesis, have been extensively studied for its molecular regulatory mechanisms at both protein and post-translational levels. However, our study sheds new light on its epigenomic regulation. In this study, we confirmed that its expression was regulated by histone modifications. Intriguingly, we identified a BRD1 binding site within the SREBF1 promoter region. BRD1 is a subunit of the MOZ/MORF acetyltransferase complex, is known to regulate multiple histone acetylation marks, including H3K9ac and H3K14ac [15]. We found that inhibition of BRD1 led to a reduction in H3K9 acetylation, but not H3K14 acetylation at the SREBF1 promoter region. Conversely, the enrichment of H3K9ac was notably decreased at the SREBF1 promoter following the deregulation of BRD1. This finding aligns with previous reports on the BRD1-interacting MOZ/MORF complex, which demonstrated MOZ’s regulation of HOX gene expression through H3K9ac in mouse embryos [35]. However, the BRD1-MOZ complex regulates embryonic stem cells differentiation of via H3K14ac [36], and reduced BRD1 expression in mouse thymocytes and brain only affected H3K14 acetylation levels [37]. These results underscore the cell-type and gene-specific regulation of histone acetylation by the BRD1 complex, which we show is also evident in the progression of HCC.

In BRD1-deficient HCC cells, H3K9ac level was decrease with an increase in H3K9me3 in BRD1 binding region of SREBF1 promoter. We observed a shift from H3K9ac to H3K9me3 within the SREBF1 gene promoter region. Concurrently, the presence of H3K14ac mark within the same promoter region of SREBF1 maintained a dual signature characterized by both H3K9me3 and H3K14ac. This combination collectively indicates an inactive state, thereby resulting in the suppression of SREBF1 expression. Notably, previous studies have identified the co-occurrence of H3K9me3 and H3K14ac in diverse human cell types, defining a poised inactive state in young liver [27, 28]. This is consistent with the BRD1-mediated regulation of SREBF1 expression we found in HCC.

Furthermore, recent study has confirmed that the H3K14ac modification partially colocalizes with H3K9me3 and its associated methyltransferase SETDB1, contributing to the reestablishment of constitutive heterochromatin in *Drosophila* early embryos during the mid-blastula transition [38]. Notably, our results corroborate that this specific epigenetic modification pattern exists to preserve a silent chromatin state, thereby suppressing SREBF1 gene expression in the absence of BRD1 in HCC cells. SETDB1 plays a cooperative role in recognizing the combinatorial histone marks H3K9me3. The SETDB1-occupancy effect may be responsible for the H3K9ac to H3K9me3 transition of the SREBF1 promoter. Collectively, the concurrent presence of H3K9me3 and H3K14ac defines an inactive state within the SREBF1 promoter region in BRD1-downregulated HCC cells. These findings contribute to a deeper understanding of the complex mechanisms underlying epigenomic regulation of SREBF1 expression in HCC.

We further conducted an in-depth examination in vivo to ascertain whether BRD1 could serve as a viable therapeutic target for HCC. Nonetheless, despite successfully inhibiting BRD1, we observed a concurrent downregulation of SREBF1, FASN and SCD1 within the HCC tumor tissue. Despite this modulation, the tumor tissue failed to exhibit a substantial reduction in size. These findings suggest that interfering with SREBF1-mediated lipid metabolism alone is insufficient to fully arrest the progression of HCC in vivo. Simvastatin, a well-established cholesterol-lowering agent, has garnered extensive use in the prophylaxis and management of cardiovascular diseases. Intriguingly, recent reports have highlighted its potential to curb the development of HCC [39]. Furthermore, simvastatin has demonstrated dual functionality in HCC by not only mitigating tumor growth but also augmenting the sensitivity of HCC cells to sorafenib through modulation of SREBP2-mediated cholesterol biosynthesis pathways [40]. In this study, we uncovered a novel therapeutic synergy between simvastatin and iBRD1 in suppressing liver cancer tumorigenesis. Specifically, our findings in a mouse xenograft model revealed that the combined administration of these two drugs potently inhibited tumor progression and reduced tumor volume. Notably, the expression levels of SREBF1, FASN and SCD1 were significantly decreased in the tumor tissues of iBRD1 and simvastatin treated animals. Collectively, these results suggest that iBRD1 and simvastatin act in concert to regulate metabolic pathways, thereby hindering the advancement of liver cancer. This will expand the use of simvastatin in HCC, especially, iBRD1 and simvastatin combination may be a good therapeutic strategy for NASH-associated HCC treatment. However, more studies are necessary to evaluate the efficacy of combinatory effects of iBRD1 and simvastatin prior to clinical investigation.

**Fig. 7:**
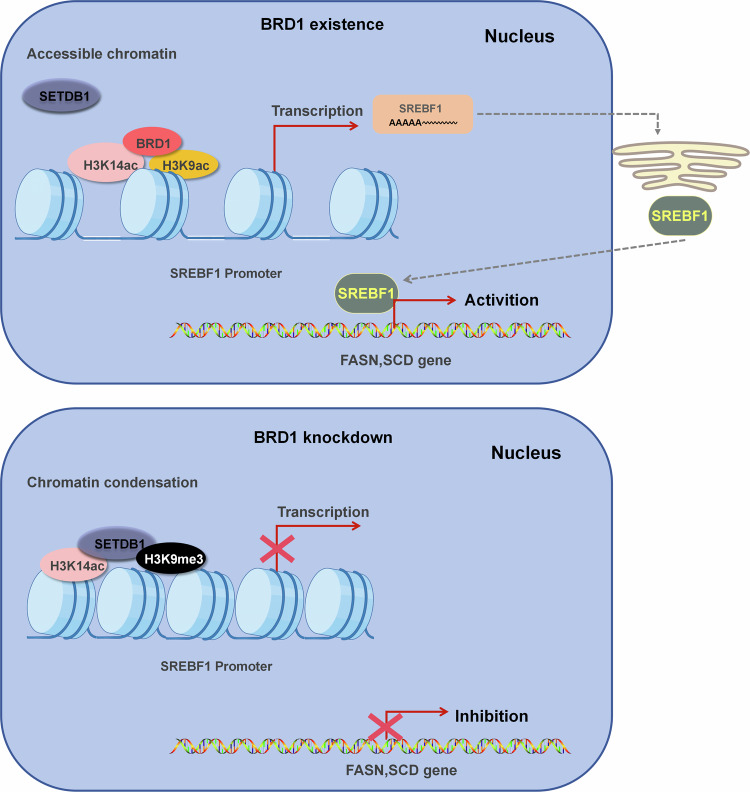
BRD1-SREBF1 axis regulates liver cancer progression model. In HCC cell, BRD1 is elevated and potentially activates SREBF1-mediated lipid metabolism via regulating the histone acetylation level of H3K9 and H3K14. Specifically, inhibiting BRD1 in HCC cells results in a decrease in the acetylation level of H3K9ac, with subsequent conversion of H3K9ac to H3K9me3. Notably, the occupancy of H3K14 at the SREBF1 promoter remains unchanged, with H3K14ac partially colocalizing with H3K9me3 and SETDB1, indicating a poised inactive state to alter SREBF1expression, ultimately inhibiting the SREBF1-FASN/SCD1 axis mediated lipid metabolism and thereby blocking HCC progression.

Based on our cumulative findings, we propose an enhanced model elucidating the BRD1-associated molecular mechanism underlying HCC progression and highlighting potential therapeutic targets. In normal hepatocytes, expression of BRD1 is minimal. However, it shows an abnormally high expression, BRD1 potentially activating SREBF1-mediated lipid metabolism via the histone acetylation of H3K9 and H3K14 in HCC. Specifically, inhibiting BRD1 in HCC cells results in a decrease in the acetylation level of H3K9ac, with subsequent conversion of H3K9ac to H3K9me3. Notably, the occupancy of H3K14ac at the SREBF1 promoter remains unchanged, with H3K14ac partially colocalizing with H3K9me3 and SETDB1, indicating a poised inactive state to alter SREBF1expression, ultimately inhibiting the SREBF1-FASN/SCD1 axis mediated lipid metabolism and thereby blocking HCC progression (Fig. 7). In conclusion, our findings contribute novel insights into the role of BRD1-associated epigenetic modifications in regulating SREBF1-directed hepatic lipid metabolism in HCC. The proposed model provides an in-depth understanding of the BRD1-ralted intricate regulatory networks governing HCC and suggests potential therapeutic strategies that target this pathway. Our work holds promise for the advancement of innovative therapeutic strategies specifically aimed at modulating fatty acid metabolism in HCC, thereby contributing to the field of molecular cancer research.

## Materials and Methods

### Cell culture and reagents

All cell lines, including HEK293-T, Huh7, HepG2, Hep3B, MHCC97-H cells, were purchased from Shanghai Zhongqiao xinzhou Biotechnology company. The HEK293-T, Huh7, MHCC97-H cells were routinely cultured in Dulbecco’s Modified Eagle Medium (DMEM, Gibco, USA), supplemented with 10% fetal bovine serum (FBS, Gibco, USA), and THLE-2, Hep3B, HepG2 were routinely cultured in Minimum Essential Media (MEM, Gibco, USA) supplemented with 10% fetal bovine serum (FBS, Gibco, USA) 1% Gluta MAX^TM^ (Gibco, USA) and 1% sodium pyruvate (Gibco, USA). All cells were incubated at 37°C in a humidified incubator with 5% CO_2_. All cell lines were confirmed by short tandem repeat (STR) analysis recently.

### RNA-seq data analysis

The RNA-seq data of shBRD1 were generated in HCC cell lines. Total RNA was extracted from both the shNC group and shBRD1 group of Huh7 cells using the RNA extraction reagent RNAiso Plus (Takara, Beijing, China). Subsequent RNA-seq sequencing was performed by Fraser (Wuhan, China) and analysis by HiSeq platform. Differentially expressed genes (DEGs) between the two groups were identified by DESeq2 method based on the read counts. Differential gene enrichment analysis was performed referencing on KEGG database. Genes significantly associated with BRD1 in LIHC were analyzed using the linked omics database (http://www.linkedomics.org/admin.php), while enrichment analysis was carried out using the Sangerbox2 tool’s enrichment analysis module (http://vip.sangerbox.com/).

### Generation of stable cell lines

Lentiviral-based small hairpin RNA (shRNA) targeting BRD1 (#1shBRD1 target sequence: GGGAGTGTCAGAACAGCAACG; #2shBRD1 target sequence: GCCACAATGAGGAAACGGTTA were obtained from Sangon Biotechnology (Shanghai, China). Primers were annealed and ligated to the pLVshRNA-EGFP(2 A) Puro vector (Miao Ling Biology, Wuhan, China). For the rescue experiments using the overexpression SREBF1 plasmid, the full-length cDNA of SREBF1 was obtained and cloned into the pReceiver-Lv242 vector (iGene Biotechnology, Guangzhou, China). Knockdown and overexpression vectors were both used in conjunction with packaging plasmids pSPAX2 and pMD2.G (Miao Ling biology, Wuhan, China) to transfect 293 T cells, in order to produce the lentiviruses. To generate stable cell lines, Huh7 and Hep3B cells were transfected with lentivirus for 24 hours, followed by selection with puromycin (4 μg/mL) for at least three days.

### Gas chromatography-mass spectrometry (GC-MS)

Cell samples from both the experimental and control groups were plated into 60 mm culture dishes at a density of 60-70%. These cultures were maintained until the target gene expression was induced. For cell counting purposes, an additional dish containing approximately 3×10^6^ cells were prepared for each group. Following cell enumeration, the culture dishes were thoroughly washed and subsequently flash-frozen using liquid nitrogen. Subsequently, 80% methanol was added, and the adherent cells were gently detached using a cell scraper. The harvested cell samples were mixed with chloroform, and following centrifugation, the supernatant was meticulously aspirated to collect the organic phase. The organic extracts were dried under a nitrogen stream and then redissolved in 2% methanol sulfuric acid. The samples were then heated at 50 °C for 2 hours to facilitate the formation of fatty acid methyl esters (FAMEs). Post-esterification, hexane and saturated sodium chloride were added to the samples. After centrifugation, the supernatant containing the FAMEs was carefully collected. Ultimately, 180 µL of each processed sample was dispensed into a micro-injection vial for gas chromatography-mass spectrometry (GC-MS) analysis using a GCMS-QP2010ULTRA system (Shimadzu, Japan). Fatty acid concentrations were determined based on the retention times of standard samples.

### Staining of lipid droplets

Cultured cells were passaged onto 12-well plate cell crawls, fixed with 4% paraformaldehyde solution (Biosharp, China), washed three times in 1×PBS, and then stained with 2 μg/mL nile red solution (HY-D0718, MCE, USA) for 10 min. The samples were then washed three times with PBS, and then stained with DAPI for 5 min, washed three times and then the images were captured using confocal microscopy.

### Quantification of cholesterol

Cholesterol levels were quantified using cholesterol kits (Bioassay Systems, Boxbio, Beijing). according to the manufacturer’s instructions. Cell samples were passage into 100 mm culture dishes to achieve a density of 60-70% and cultured overnight. Following trypsinization without EDTA, the cells were centrifuged to remove the supernatant and subsequently resuspended in 4 mL of PBS. 1 mL aliquot of the cell suspension was combined with 50 µL of protein lysis buffer, and the protein concentration was quantified using the BCA method. The remaining 3 mL of cell sample was centrifuged, resuspended in 300 µL of isopropanol, and transferred to a 1.5 mL centrifuge tube. Cells were sonicated on ice at full power for intervals set at 2 seconds on followed by a pause of 3 seconds, totaling a sonication duration of 5 min. The samples were then centrifuged at 4°C at an acceleration force of 10,000 g for 10 min; the supernatant was placed on ice for subsequent analysis. The cholesterol detection working solution was freshly prepared in a ratio comprising reagent one: reagent two: reagent three = 1 mL:10 µL:10 µL (Bioassay Systems, Boxbio, Beijing). In a standard flat-bottomed microplate (96-well plate), either test samples or controls (blank group or standard group) each received an addition of 20 µL followed by adding 180 µL of the cholesterol detection working solution derived from cells. After thorough mixing, plates were incubated in darkness at 37 °C for 15 min before measuring absorbance at 500 nm. Cholesterol concentrations were calculated based on the standard curve equation. Concurrently, the protein concentration of an addition alone fourth portion of the cells was determined using the BCA method. Total cholesterol (µg/mg protein) = 386.654 × cholesterol concentration (µmol/mL) /protein concentration (µg/µL).

### Detection of cell proliferation by CCK8 assay

Cells were counted and plated in 96-well plates at a density of 1000 cells per well, with three replicates per group. The CCK8 assay was initiated 24 hours post-plating by adding 10 μL of CCK8 (Beyotime Biotechnology, Shanghai, China) solution to each well containing 100 μL of complete medium. Absorbance values at 450 nm were measured using an enzyme marker after 3 hours, and the assay was repeated for four consecutive days. Cell proliferation curves were generated based on the assay data.

### Edu detection of cellular nascent DNA

The EdU-647 Cell Proliferation Detection Kit (as per manufacturer’s instructions) was utilized to assess cell proliferation. Briefly, the Edu solution (Beyotime Biotechnology, Shanghai, China) was diluted in culture medium and added to cellular monolayers in 12-well plates at a concentration of 1×. After a 2-hour incubation period, the cells were treated with 1× Hoechst 33342 for an additional 10 min at room temperature, ensuring protection from light. Following this, the cells were washed three times with washing solution. Images were captured using a laser confocal microscope, and the proportion of Edu-positive cells was quantified.

### Clone formation assay

Cells were collected and counted, and inoculated into 6-well plates at densities of 8000 or 2000 cells per well, with three replicates per group. After 14 days of culture, the medium was discarded, and the cells were stained with 0.1% crystal violet solution for 1 hour at room temperature, protected from light. Excess stain was removed with pure water, and the stained clones were counted after drying.

### Cell cycle assay

Cell cycle assay was performed using a Cytoflex LX flow cytometer (Beckman Coulter) according to the DNA Content Assay Kit protocol (cat. no. CA1510, Solarbio, China). Cells from both the experimental and control groups were initially plated into 60 mm culture dishes and allowed to grow to approximately 90% confluence. Subsequently, the cells were trypsinized and collected into 15 mL centrifuge tubes. Subsequently, the collected cells were then resuspended and thoroughly washed with pre-chilled PBS. Following this, the cells were fixed in 70%-75% ethanol at −20°C for a minimum of 2 hours. After fixation, the cells were retrieved from −20°C and washed again with PBS. To ensure a uniform cell suspension, the cells were filtered through a 200-mesh sieve. Next, to eliminate RNA contamination, the cells were treated with RNase A. Finally, propidium iodide (PI) dye was added, and the cells were incubated in the dark at room temperature for at least 20 min. The red fluorescence intensity was then measured at 488 nm using a flow cytometer.

### Apoptosis assay

Apoptosis was evaluated using the Annexin V Alexa Fluor 647/PI Apoptosis Detection Kit (CA1050, Solarbio, Beijing, China) on a Cytoflex LX flow cytometer (Beckman Coulter, Beijing, China). Cells were digested and plated into 60 mm culture dishes at an inoculation density of 50-70%. These cells were then cultured overnight to allow for adherence and growth. Subsequently, the probe binding buffer was diluted with deionized water at a ratio of 1:9, with 1.5 mL of the diluted buffer being prepared per sample based on the amount required. After collecting the cell culture medium, the cells were gently detached using low-concentration trypsin without EDTA, which was added until the cells could be easily dislodged with a minimal pipetting motion. The detached cells were then dispersed and collected into a centrifuge tube, followed by centrifugation for 5 min at an appropriate speed to sediment the cells. Following centrifugation, PBS was added to resuspend the cells, and the suspension was centrifuged once again to remove any remaining supernatant. The cells were then resuspended in the previously diluted binding buffer and transferred to flow cytometry tubes or 1.5 mL centrifuge tubes. Next, 7 μL of Annexin V-Alexa Fluor 647 was added to each tube, and the contents were thoroughly mixed and incubated in the dark at room temperature for 5 min. Subsequently, 5 μL of propidium iodide and an additional volume of pre-chilled PBS were added to each tube. The stained cell samples were analyzed using a flow cytometer within 1 hour of staining. Based on the cell morphology observed in the flow cytometry scatter plot, the positive staining range was determined, and gates were drawn to record cell data at a low acquisition speed (10 μL/s) to ensure accuracy and precision.

### Wound-healing assays

To assess cell migration, a wound-healing assay was conducted in 6-well plates. Each well was seeded with a 4×10^5^ cell suspension and allowed to adhere overnight. A scratch was made using a 20 μL pipette tip, and the detached cells were removed. The cells were then incubated in serum-free medium. Wound closure was monitored at 24 and 48 hours or 72 hours post-scratch, and the remaining cell-free area was measured following image acquisition.

### Quantitative real-time RT-PCR (qRT-PCR)

Total RNA was extracted from HCC cells (shNC and shBRD1 groups) using RNAiso Plus (Takara, Beijing, China). Reverse transcription was performed using the Prime Script™ RT reagent Kit (Takara). qRT-PCR was conducted on a Step One Plus real-time PCR system (Thermo Fisher Scientific) using TB Green® Premix Ex Taq™ II (Tli RNaseH Plus) (Takara). GAPDH served as the internal control for normalizing target gene expression levels, calculated using the formula 2 (2^-ΔΔCt^). Primer sequences are listed in Supplementary Table 1.

### Western Blotting

Protein concentrations were determined using the Bio-Rad Protein Assay Kit. Protein lysates were prepared from HCC cells using Laemmli sample buffer (Bio-Rad). SDS-PAGE followed by transfer to PVDF membranes was performed. Primary antibodies were incubated overnight, and secondary antibodies were incubated for 1 hour at room temperature. Membranes were developed using an ECL kit. Primary antibody details are provided in Supplementary Table 2.

### ChIP-qPCR assays

A ChIP assay was performed using the Simple ChIP Enzymatic Chromatin IP Kits (catalog number 9005S, Cell Signaling Technology, China), in strict accordance with the manufacturer’s instructions. The cells at 80% confluence were fixed with formaldehyde (final concentration of 1%, directly added to the culture media) for a duration of 10 minutes. Then, the cells were centrifuged and lysed in 200 μl of membrane extraction buffer supplemented with a protease inhibitor cocktail. To obtain chromatin fragments, cell lysates underwent micrococcal nuclease digestion for 30 minutes at 37 °C, followed by sonication (comprising three cycles of 20 seconds on and 30 seconds off at an amplitude of 35%) to generate DNA fragments ranging from 100 to 500 base pairs in length. After centrifugation, the clarified supernatant was diluted in a chip buffer (to a final volume of 500 µL) containing a protease inhibitor cocktail. A 10 µL aliquot was set aside as a 2% Input. The remaining solution was incubated overnight at 4°C with either the primary antibody, as shown in Supplementary Table 2 or an appropriate normal IgG antibody on a rotator. The following day, immunoprecipitation reactions were further incubated for an additional two hours with ChIP-Grade Protein G Magnetic Beads. After bead precipitation, beads were washed sequentially with low and high salt solutions. Chromatin elution from Antibody/Protein G Magnetic beads and reversal of cross-linking were achieved through heat treatment (65°C, 30 min). The purified DNA was subsequently obtained using spin columns and analyzed via SYBR Green-based real-time PCR. The modification changes of the promoter were assessed by calculating the ratio relative to the Input. Subsequently, the qPCR products were loaded onto a 3% agarose gel for electrophoresis to ascertain the specificity of the bands. Primer sequences targeting the SREBF1 promoter are provided. (SREBF1-F:GGAGACAAAGGCCAGGGAGA；SREBF1-R: TGACCGACATCGAAGGTGC.)

### In vivo xenograft model

Male BALB/c nude mice (5 weeks old) were obtained from Vital River Laboratory Animal Technology (Beijing). Mice were randomized into two groups (n = 6 per group) and subcutaneously injected with HCC cells or BRD1-downregulated HCC cells (5 × 10^6^ cells per mouse). Tumor growth was monitored every 4 days using a caliper, and tumor volume was calculated using the formula: volume (cm) = L × W × 0.5. After 25 days, mice were euthanized, and tumors were harvested for analysis, The collected tumors were fixed, embedded, and sectioned, after which IHC was performed to detect the expression of PCNA protein in the tumor samples.

In the mouse model of xenograft tumors treated with pharmacological agents, the xenograft tumor model was initially established using the aforementioned method. Seven days post-establishment, the mice were randomly assigned to four groups, each consisting of six mice. Administration was conducted daily via the intraperitoneal route. The treatment groups received either saline, NI-57 (MCE, USA) at a dosage of 10 mg/kg, simvastatin (MCE, USA) at a dosage of 40 mg/kg, or a combination of both NI-57 and simvastatin. Tumor volumes were recorded every two days. After 12 days, the mice were euthanized for analysis, and their weights were measured. Subsequently, IHC was performed to detect the expression of SREBF1, FASN, and SCD1 proteins level in the tumor samples. All animal experiments adhered to the guidelines approved by the Experimental Animal Ethics Committee of the School of Life Sciences, Inner Mongolia University. Sample size was determined based on achieving statistically significant results.

### Data analysis

All statistical analyses were performed using GraphPad Prism 8 (GraphPad Software v8.0.2.236 San Diego, CA). Differences between the two groups were analyzed using a two-tailed unpaired Student’s t-test. Data were presented as Mean ± SD as indicated in the figure legends and the significance levels are indicated as * *p* < 0.05, ** *p* < 0.01, *** *p* < 0.001, ****p < 0.0001, or ns (not significant) *p* values of < 0.05 were considered statistically significant. All experiments were performed at least in triplicate. Furthermore, we performed a quantitative analysis of the data and conducted statistical evaluations on the results obtained from various biological replicates for each Western Blot experimental chart.

## Supplementary information

Supplementary figure and table

Original data

## Data Availability

All data is already released and could obtain from the corresponding authors.
