# Supplementary material for: BRD1 deficiency affects SREBF1-related lipid metabolism through regulating H3K9ac/H3K9me3 transition to inhibit HCC progression
Source: Cell Death Dis. 2025 Feb 17;16(1):104. doi: 10.1038/s41419-025-07404-7 (PMC11833140; doi:10.1038/s41419-025-07404-7)
Supplement: Supplementary file 1 — Supplementary figure and table [file 41419_2025_7404_MOESM1_ESM.pdf]

Supplementary figure and table legends

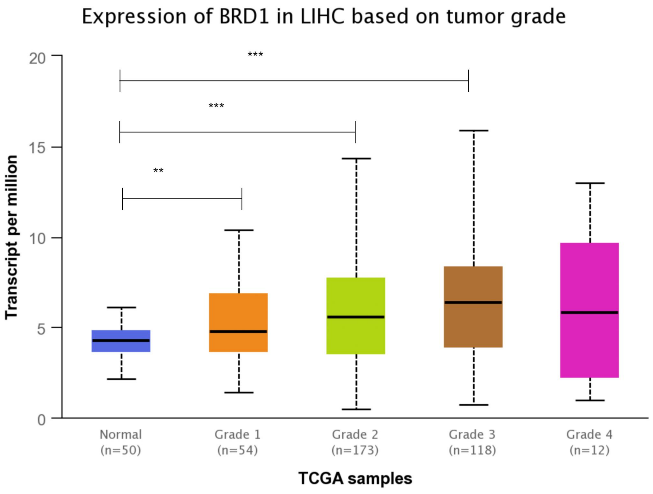

Figure S1. Analysis of BRD1 expression in clinical samples of HCC patients.

Correlation analysis of BRD1 expression and clinical grade in HCC patients, all data were sourced from TCGA database (http://ualcan.path.uab.edu/).

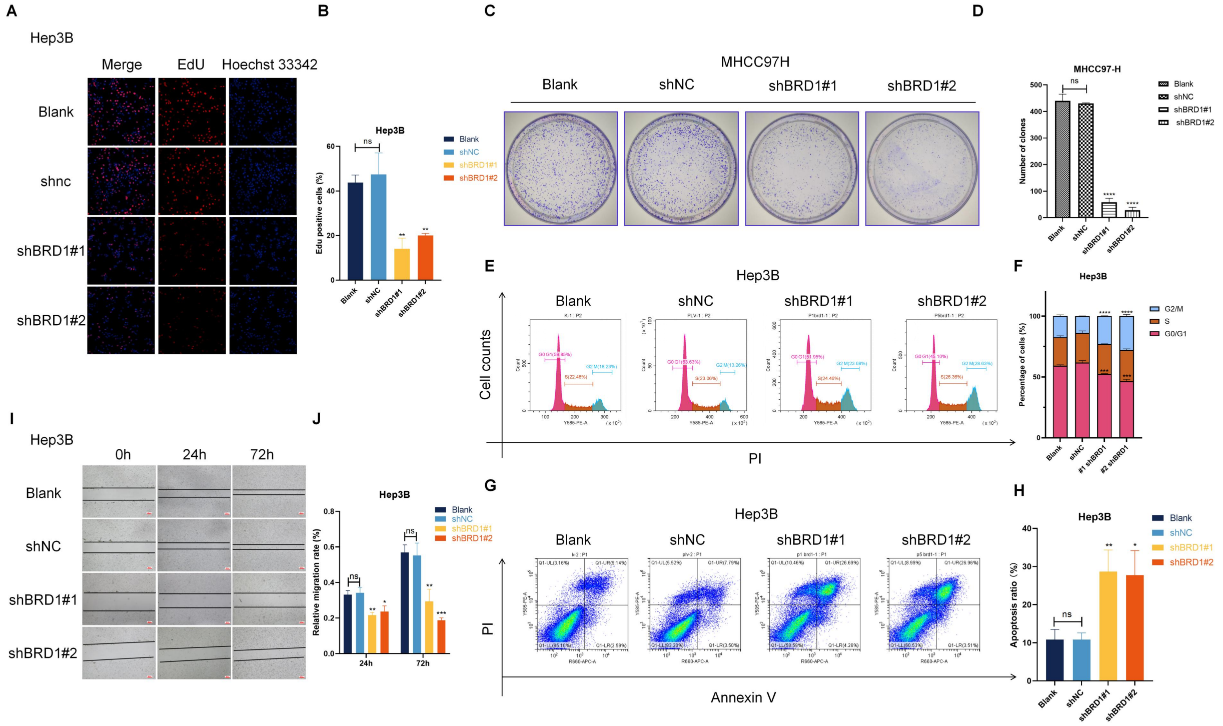

Figure S2. BRD1 promotes HCC cell growth and migration in Hep3B cells.

(a) The detection of Edu-labeled DNA was performed using a confocal microscope. (b) Cell viability rates after BRD1 downregulation as compared to the controls. (c, d) Colony-forming ability of MHCC97H cells analyzed by clone formation assay. (e, f) The cell cycle was determined

after BRD1 downregulation in Hep3B cells. Representative images for cell cycle assays in indicated cell lines, and statistical analysis in the right panel. (g, h) The cell apoptosis was determined by analyzing Annexin V-FITC PI in BRD1 downregulated Hep3B cells. (i, j) HCC cell transfected with BRD1 shRNA showed lower motility in a wound healing assay, compared with control cells. Data are presented as the mean ± SD from three independent experiments. ns $p > 0.05$; *$p < 0.05$; **$p < 0.01$; ***$p < 0.001$; ****$p < 0.0001$.

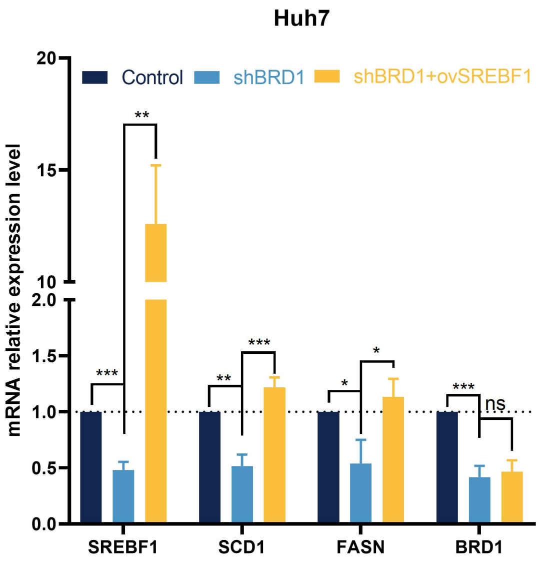

Figure S3. the expression levels of the FASN and SCD1 genes were elevated following the overexpression of SREBF1 in BRD1 knockdown HCC cells.

The qPCR shows mRNA level of SREBF1, FASN, and SCD1 in HCC cells transfected with control vector, shBRD1 vector, or shBRD1 vector in combined with SREBF1-overexpressing vector. Data are presented as the mean ± SD from three independent experiments. ns $p > 0.05$; *$p < 0.05$; **$p < 0.01$; ***$p < 0.001$.

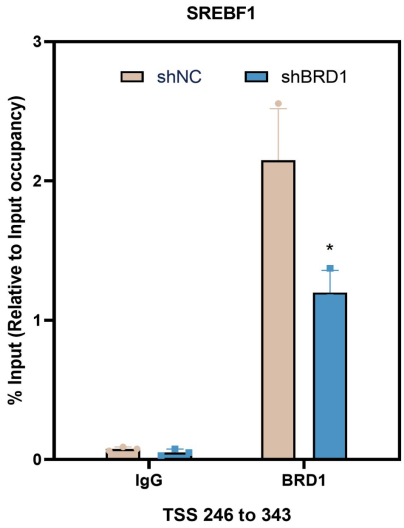

Figure S4. BRD1 enrichment at the SREBF1 promoter was reduced upon BRD1 knockdown.

The enrichment level of BRD1 at the SREBF1 promoter were detected using ChIP-qPCR. Data are presented as the mean ± SD from three independent experiments. $*p < 0.05$.

Table S1 Primer sequences

Gene	Primer sequences
GAPDH-F	GCACCGTCAAGGCTGAGAAC
GAPDH-R	TGGTGAAGACGCCAGTGG
SREBF1-F	ACTTCTGGAGGCATCGCAAGCA
SREBF1-R	AGGTTCCAGAGGAGGCTACAAG
FASN-F	ACAGCGGGGAATGGGTACT
FASN-R	GACTGGTACAACGAGCGGAT
SCD1-F	TCTAGCTCCTATACCACCACCA
SCD1-R	TCGTCTCCAACTTATCTCCTCC
ACSL4-F	GCTATCTCCTCAGACACACCGA
ACSL4-R	AGGTGCTCCAACTCTGCCAGTA
CD36-F	CAGGTCAACCTATTGGTCAAGCC
CD36-R	GCCTTCTCATCACCAATGGTCC
CPT1A-F	GATCCTGGACAATACCTCGGAG
CPT1A-R	CTCCACAGCATCAAGAGACTGC

Supplementary Table S1. Gene expression detection quantitative PCR primer sequence.

Table S2 Main antibody information

Antibody name	Percentage of dilution	Experiment	Source
GAPDH	1:5000	WB	Proteintech (China)
BRD1	1:1500	WB	Abcam（USA）
H3K14ac	1:2000	WB	Abcam（USA）
SETDB1	2.0 μg/IP	Chip	Abcam（USA）
H3K9me3	2.0 μg/IP	Chip	Abcam（USA）
H3K9ac	2.0 μg/IP	Chip	Abcam（USA）
H3	1:1000	WB	CST（USA）
SREBF1	1:5000	WB/ Chip	Proteintech (China)
FASN	1:1000	WB/ IHC	Proteintech (China)
SCD1	1:1000	WB/IHC	Proteintech (China)
PCNA	1:10000	IHC	Proteintech (China)

Supplementary Table S2. Antibody information of Western blot and ChIP experiment.
