# Supplementary material for: BRD1 deficiency affects SREBF1-related lipid metabolism through regulating H3K9ac/H3K9me3 transition to inhibit HCC progression
Source: Cell Death Dis. 2025 Feb 17;16(1):104. doi: 10.1038/s41419-025-07404-7 (PMC11833140; doi:10.1038/s41419-025-07404-7)

WB RAW DATA:
Fig. 1B

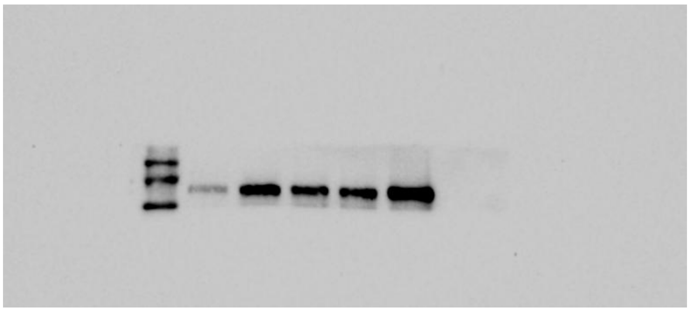

Antibody: BRD1
Lane1: Maker; Lane2-6: THLE-2, Hep3B, HepG2, MHCC97H, Huh7

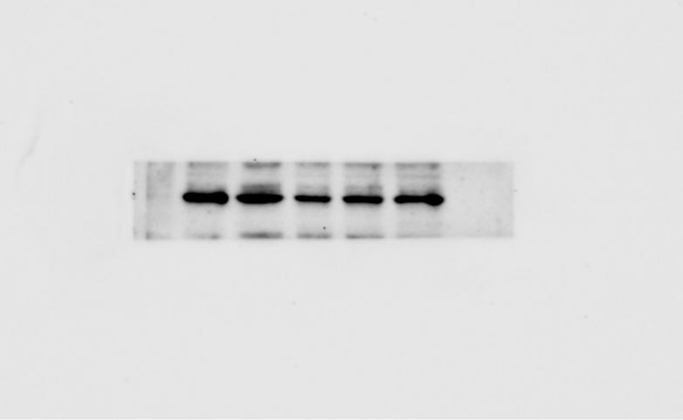

Antibody: β-actin
Lane1: Maker; Lane2-6: THLE-2, Hep3B, HepG2, MHCC97H, Huh7

Fig. 1E

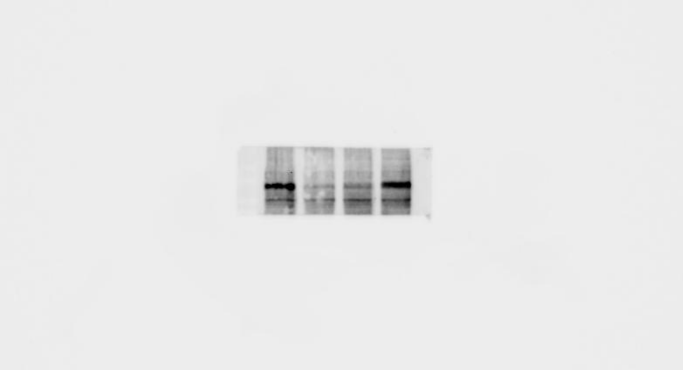

Antibody:BRD1
Lane1-4:shNC,shBRD1#1,shBRD1#2,shNC

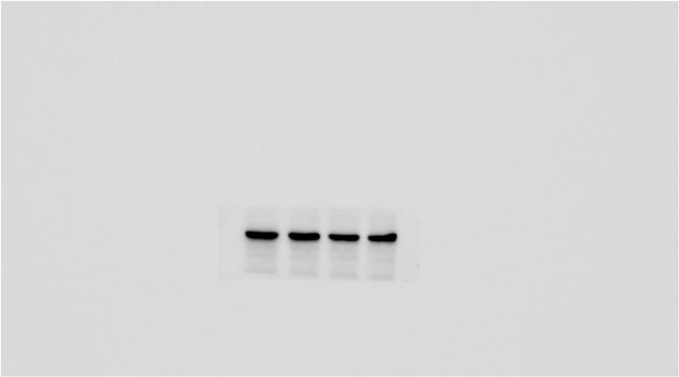

Antibody: GAPDH

Lane1-4: shNC, shBRD1#1, shBRD1#2, shNC

Fig. 3B-Huh7

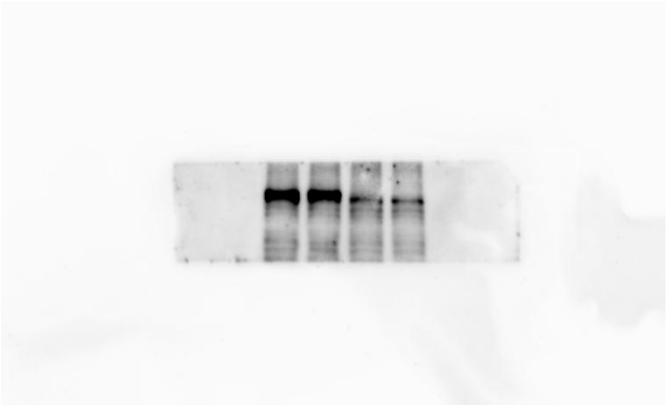

Antibody: BRD1

Lane1: Maker; Lane2-5: Blank, shNC, shBRD1#1, shBRD1#2

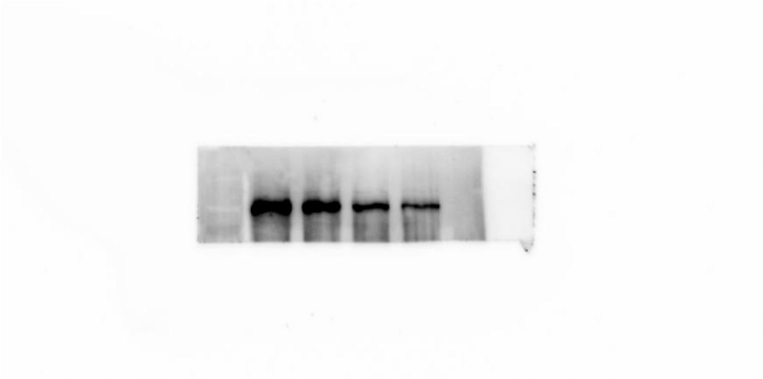

Antibody: SREBF1

Lane1: Maker; Lane2-5: Blank, shNC, shBRD1#1, shBRD1#2

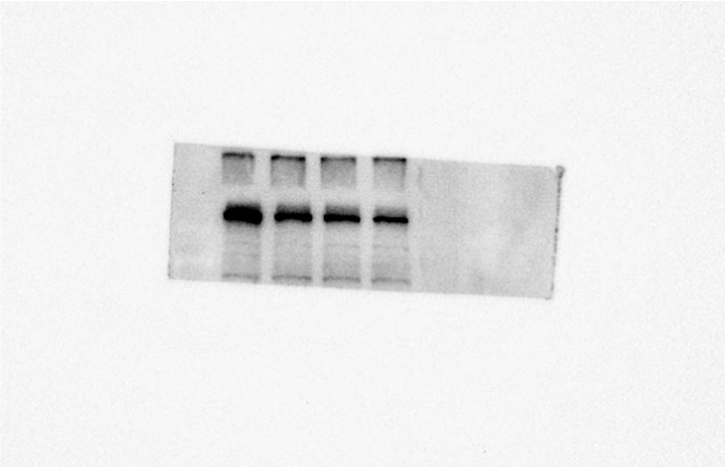

Antibody: FASN

Lane1: Maker; Lane2-5: Blank, shNC, shBRD1#1, shBRD1#2

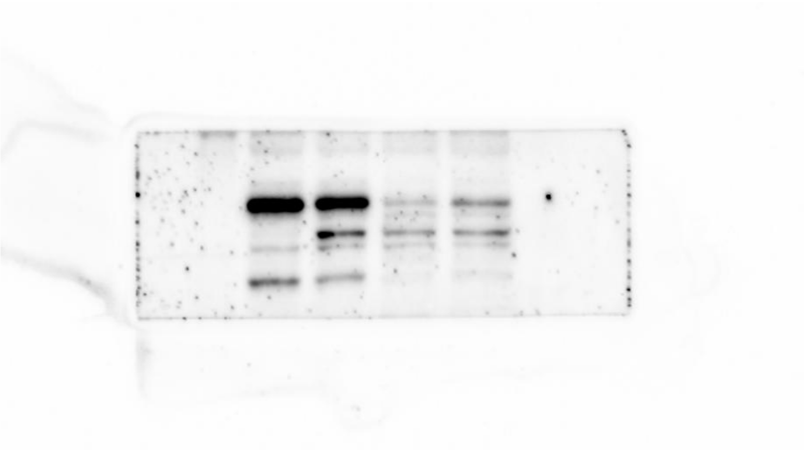

Antibody:SCD1

Lane1: Maker; Lane2-5: Blank, shNC, shBRD1#1, shBRD1#2

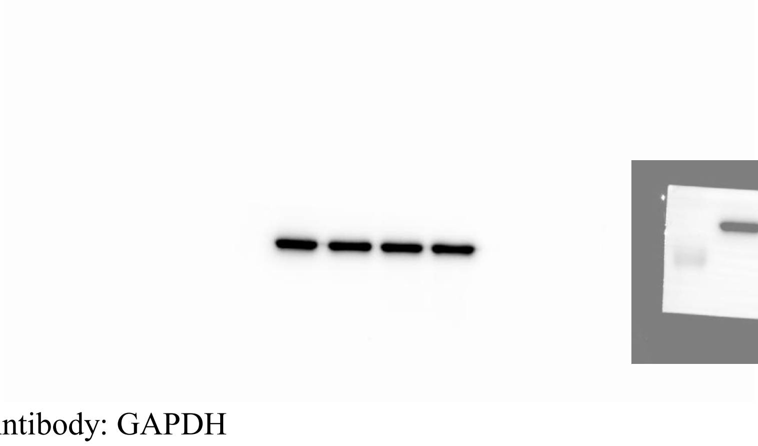

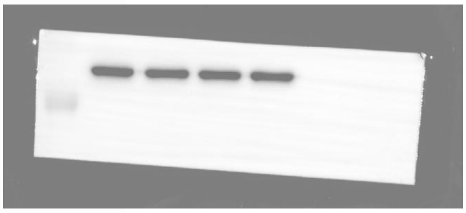

Antibody: GAPDH

Lane1: Maker; Lane2-5: Blank, shNC, shBRD1#1, shBRD1#2

Fig. 3B-Hep3B

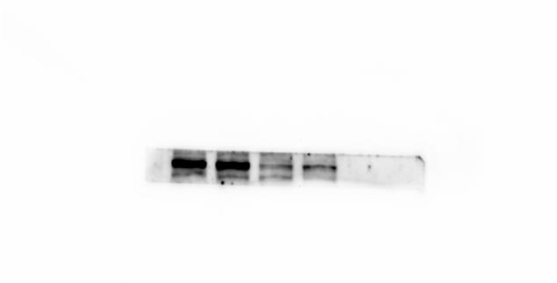
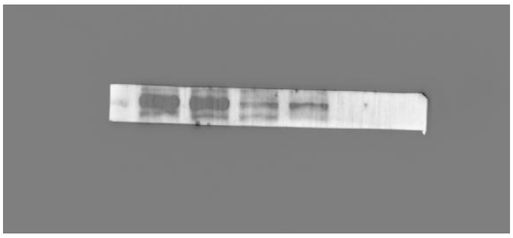

Antibody: BRD1
Lane1: Maker; Lane2-5: Blank, shNC, shBRD1#1, shBRD1#2

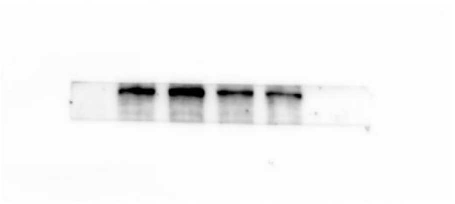
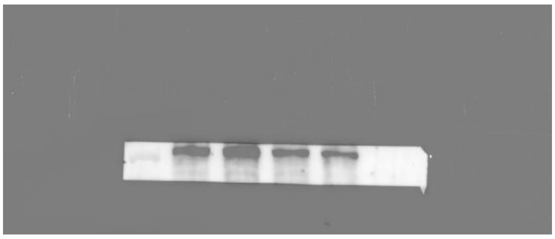

Antibody: SREBF1
Lane1: Maker; Lane2-5: Blank, shNC, shBRD1#1, shBRD1#2

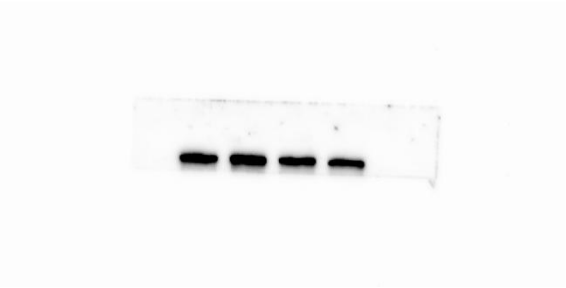
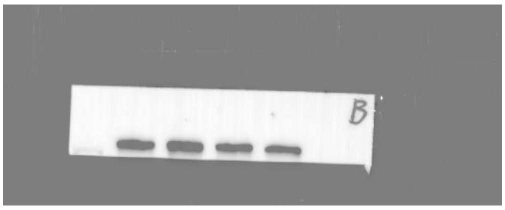

Antibody: FASN
Lane1: Maker; Lane2-5: Blank, shNC, shBRD1#1, shBRD1#2

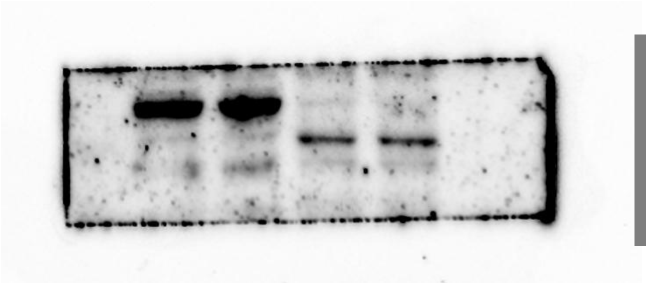
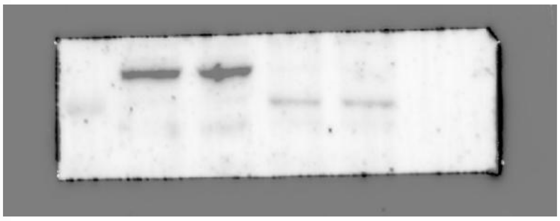

Antibody:SCD1
Lane1: Maker; Lane2-5: Blank, shNC, shBRD1#1, shBRD1#2

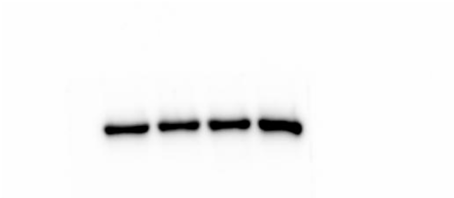
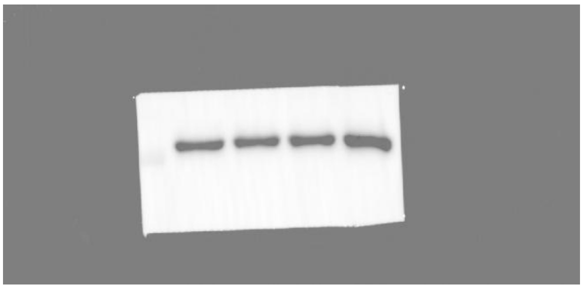

Antibody: GAPDH
Lane1: Maker; Lane2-5: Blank, shNC, shBRD1#1, shBRD1#2

Fig. 3-I

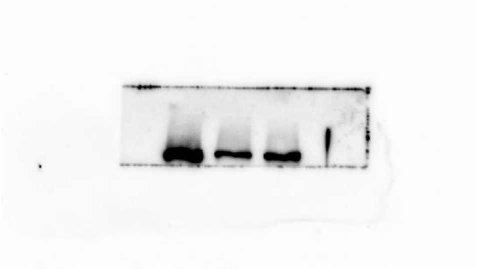
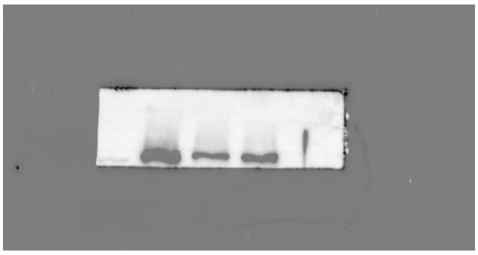

Antibody: FASN
Lane1: Maker; Lane2-4: shNC+ovNC, shBRD1#1,shBRD1+ovSREBF1

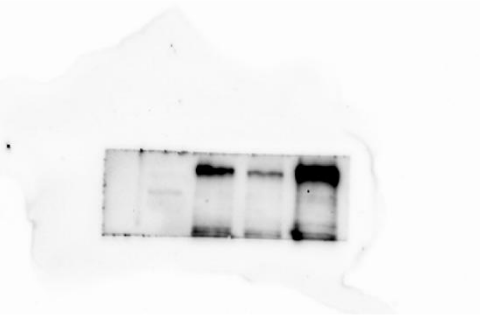

Antibody: SREBF1
Lane1: Maker; Lane2-4: shNC+ovNC, shBRD1#1, shBRD1+ovSREBF1

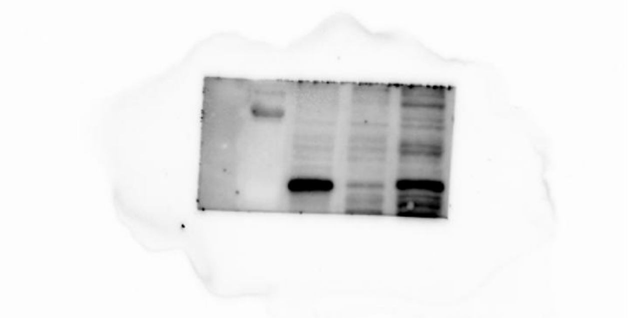

Antibody:SCD1
Lane1: Maker; Lane2-4: shNC+ovNC, shBRD1#1, shBRD1+ovSREBF1

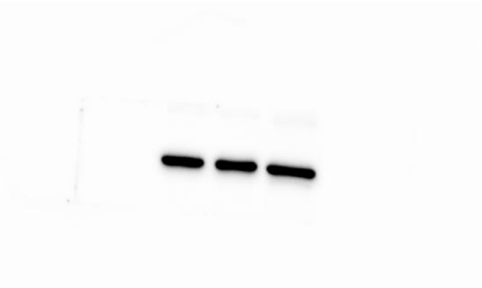

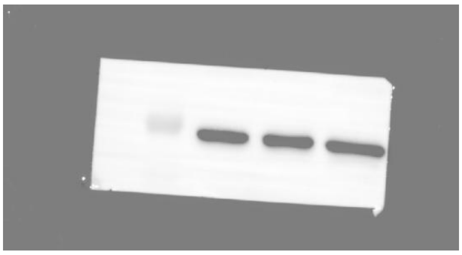

Antibody: GAPDH
Lane1: Maker; Lane2-4: shNC+ovNC, shBRD1#1, shBRD1+ovSREBF1

Fig. 3-J

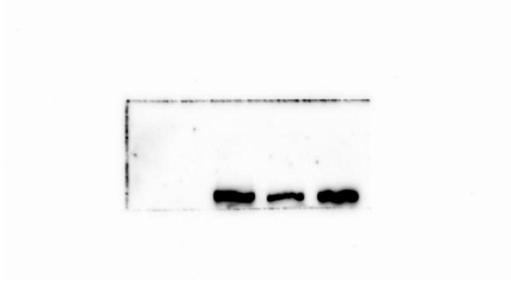

Antibody: FASN

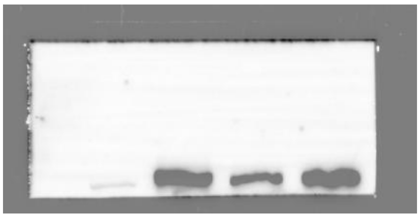

Lane1: Maker; Lane2-4: shNC+ovNC, shBRD1#1, shBRD1+ovSREBF1

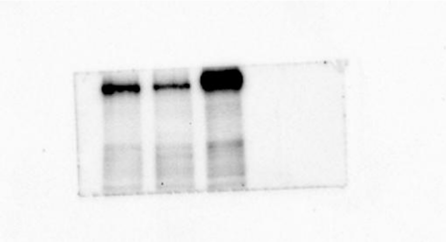

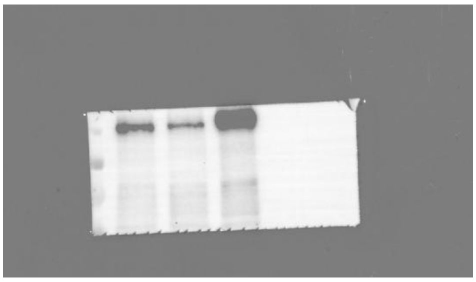

Antibody: SREBF1
Lane1: Maker; Lane2-4: shNC+ovNC, shBRD1#1, shBRD1+ovSREBF1

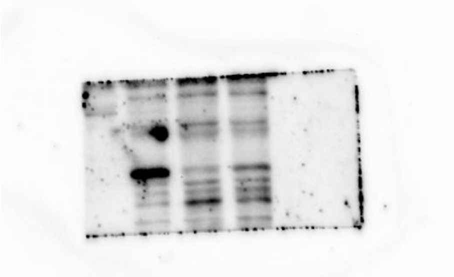

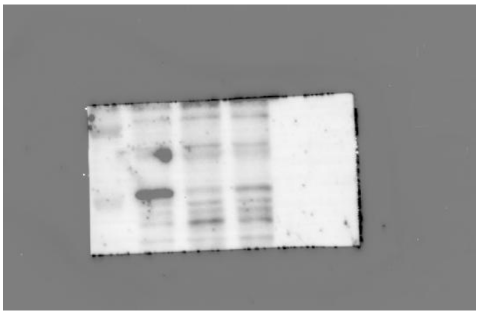

Antibody:SCD1
Lane1: Maker; Lane2-4: shNC+ovNC, shBRD1#1, shBRD1+ovSREBF1

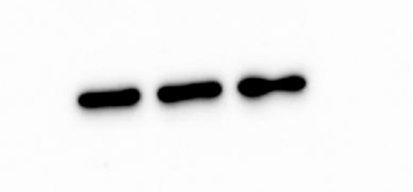

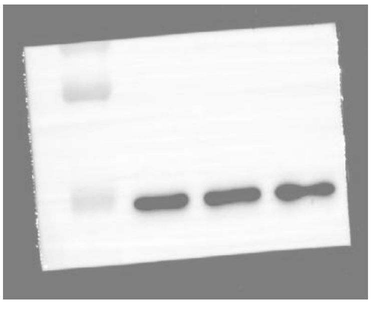

Antibody: GAPDH
Lane1: Maker; Lane2-4: shNC+ovNC, shBRD1#1, shBRD1+ovSREBF1

Fig. 5-A-Huh7

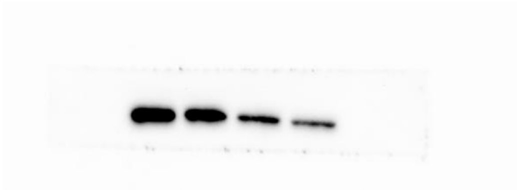
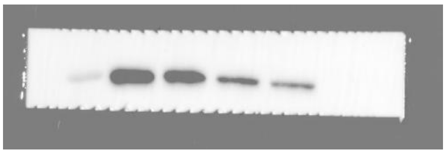

Antibody:H3K14ac
Lane1: Maker; Lane2-5: Blank, shNC, shBRD1#1, shBRD1#2

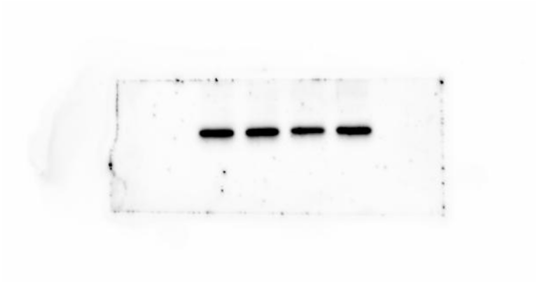
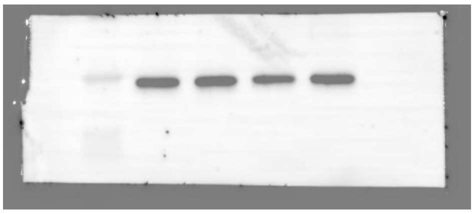

Antibody:H3K9ac
Lane1: Maker; Lane2-5: Blank, shNC, shBRD1#1, shBRD1#2

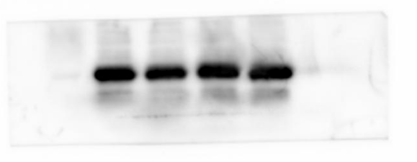
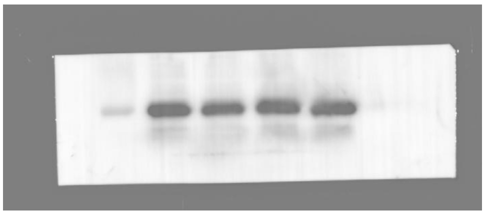

Antibody:H3
Lane1: Maker; Lane2-5: Blank, shNC, shBRD1#1, shBRD1#2

Fig. 5-A-Hep3B

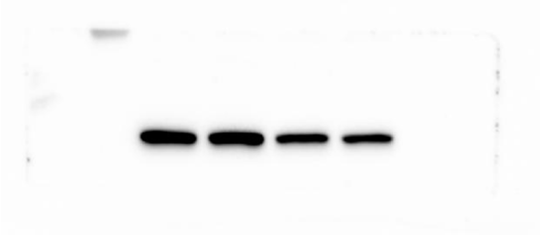
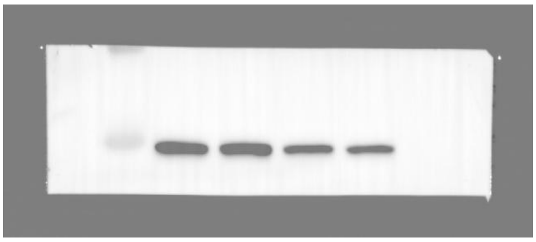

Antibody: H3K14ac
Lane1: Maker; Lane2-5: Blank, shNC, shBRD1#1, shBRD1#2

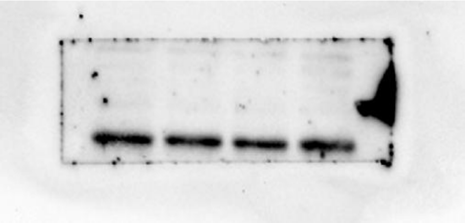

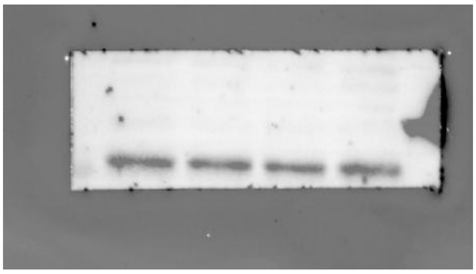

Antibody: H3K9ac
Lane1: Maker; Lane2-5: Blank, shNC, shBRD1#1, shBRD1#2

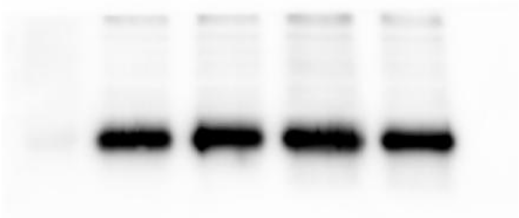

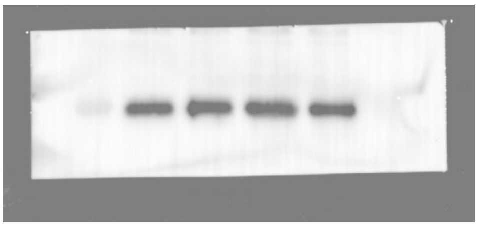

Antibody: H3
Lane1: Maker; Lane2-5: Blank, shNC, shBRD1#1, shBRD1#2

Figure 1B Western blot optical density ratio and statistical results

	THLE-2	Hep3B	HepG2	MHCC97H	Huh7
repeat1	0.12931219	0.682330917	1.020689328	0.846212296	1.159333843
repeat2	0.224893207	0.631749725	0.846915473	0.6360713	1.004512001
repeat3	0.259035268	0.689758475	0.48264158	0.696739435	1.014577688
mean	0.204413555	0.667946372	0.78341546	0.72634101	1.059474511
SD	0.054903439	0.02577389	0.224199366	0.088306283	0.070730683

Figure 1E Western blot optical density ratio and statistical results

repeat1	0.92848145	0.43573949	0.462465352
repeat2	0.872914992	0.290707562	0.485573388
repeat3	0.882254209	0.468251394	0.314800247
mean	0.894550217	0.398232815	0.420946329
normalization	1	0.445176698	0.470567692
SD	0.024294055	0.077181673	0.075647156

Figure 3B Western blot optical density ratio and statistical results
Huh7

BRD1	Blank	shNC	shBRD1#	shBRD1#2
repeat1	1.20854	1.17304	0.55816	0.2939731
repeat2	1.09311	1.10094	0.49466	0.5982699
repeat3	1.22435	1.17838	0.47407	0.47885
mean	1.17533	1.15078	0.50896	0.457031
normalization(/shNC)	1.02133	1	0.44228	0.3971475
SD	0.0585	0.03531	0.03579	0.125183

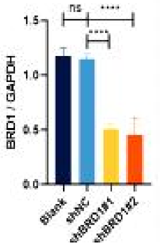

SREBF1	Blank	shNC	shBRD1#	shBRD1#2
repeat1	1.3778	0.87849	0.63308	0.7139601
repeat2	1.03262	0.96695	0.60991	0.5146208
repeat3	0.95002	0.93105	0.62592	0.5733246
mean	1.12015	0.92549	0.62297	0.6006352
normalization(/shNC)	1.21032	1	0.67312	0.648988
SD	0.18528	0.03633	0.00969	0.0836398

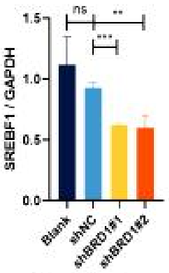

FASN	Blank	shNC	shBRD1#	shBRD1#2
repeat1	1.26025	0.87404	0.74971	0.5919506
repeat2	1.07202	0.90053	0.78469	0.6874905
repeat3	0.91003	0.81063	0.7601	0.6205641
mean	1.08076	0.86173	0.76483	0.6333351
normalization(/shNC)	1.25417	1	0.88755	0.7349543
SD	0.14311	0.03772	0.01467	0.0400357

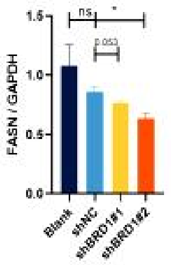

SCD1	Blank	shNC	shBRD1#	shBRD1#2
repeat1	1.23129	0.78053	0.10334	0.1410862
repeat2	1.15519	1.00967	0.18386	0.2867929
repeat3	1.06205	1.10792	0.21508	0.2171807
mean	1.14951	0.96604	0.16743	0.2150199
normalization(/shNC)	1.18992	1	0.17331	0.2225787
SD	0.06921	0.13717	0.04707	0.0595041

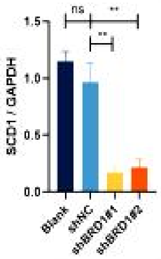

Figure 3B Western blot optical density ratio and statistical results
Hep3B

BRD1	Blank	shNC	shBRD1#1	shBRD1#2
repeat1	1.046199	0.852512	0.406988752	0.47323
repeat2	0.96702	0.850461	0.45191511	0.450026
repeat3	1.116441	1.115507	0.280603814	0.27363
mean	1.04322	0.939493	0.379835892	0.398962
normalization(/shNC)	1.110408	1	0.404298799	0.424657
SD	0.061037	0.124463	0.072525161	0.089128

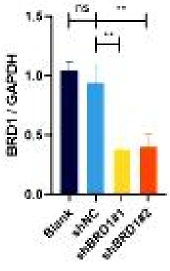

SREBF1	Blank	shNC	shBRD1#1	shBRD1#2
repeat1	0.801199	0.852512	0.510289	0.47323
repeat2	0.83202	0.850461	0.594392	0.360026
repeat3	0.880441	1.115507	0.399604	0.24363
mean	0.837887	0.939493	0.501428333	0.358962
normalization(/shNC)	0.89185	1	0.533722267	0.382081
SD	0.032615	0.124463	0.079768309	0.093737

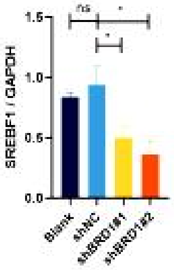

FASN	Blank	shNC	shBRD1#1	shBRD1#2
repeat1	1.215309	1.001736	0.835312396	0.812722
repeat2	0.993039	0.889677	0.73393254	0.693284
repeat3	1.138803	1.087129	0.914302562	0.87794
mean	1.115717	0.992847	0.827849166	0.794649
normalization(/shNC)	1.123755	1	0.833813433	0.800374
SD	0.092198	0.080854	0.073824617	0.076461

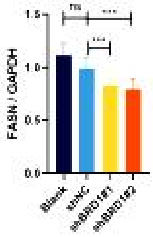

SCD1	Blank	shNC	shBRD1#1	shBRD1#2
repeat1	1.372962	1.007595	0.075084638	0.083675
repeat2	0.794533	0.687529	0.059146274	0.100842
repeat3	1.125634	1.099659	0.245148241	0.249314
mean	1.09771	0.931594	0.126459718	0.14461
normalization(/shNC)	1.178314	1	0.135745526	0.155229
SD	0.236967	0.176626	0.084177321	0.074368

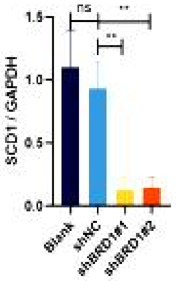

Figure 3I Western blot optical density ratio and statistical results Huh7

FASN	shNC+ovNC	shBRD1	shBRD1+ovSREBF1
repeat1	0.965119867	0.496054868	0.699223591
repeat2	0.916221835	0.604356626	0.715328494
repeat3	0.830693365	0.610867726	0.76489852
mean	0.904011689	0.570426407	0.726483535
normalization(/shNC)	0.999999656	0.630994286	0.803621561
SD	0.055554399	0.052655756	0.027947871

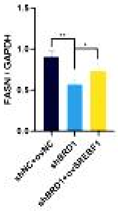

SREFB1	shNC+ovNC	shBRD1	shBRD1+ovSREBF1
repeat1	0.457326799	0.174813893	0.836135232
repeat2	0.596808706	0.31990313	0.897534349
repeat3	0.497465931	0.227602706	0.9949044
mean	0.517200479	0.240773243	0.90952466
normalization(/shNC)	0.999999959	0.465531729	1.758553328
SD	0.05862815	0.059960092	0.065369401

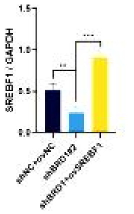

SCD1	shNC+ovNC	shBRD1	shBRD1+ovSREBF1
repeat1	0.879369848	0.269862284	0.994460532
repeat2	1.022661691	0.402634497	1.019176825
repeat3	0.763250281	0.360478365	1.045042004
mean	0.888427273	0.344325049	1.019559787
normalization(/shNC)	1.000000308	0.387567069	1.147601083
SD	0.106097746	0.055394418	0.020651575

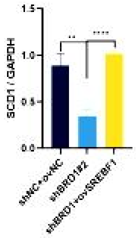

Figure 3J Western blot optical density ratio and statistical results Hep3B

FASN	shNC+ovNC	shBRD1	shBRD1+ovSREBF1
repeat1	1.228599212	0.686225137	1.104819695
repeat2	0.91570029	0.557708887	0.829476788
repeat3	1.143080648	0.70121163	0.871566273
mean	1.095793383	0.648381885	0.935287585
normalization(/shNC)	1.00000035	0.591701065	0.853525789
SD	0.132044179	0.064406745	0.121102528

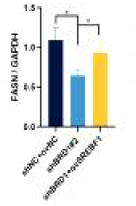

SREFB1	shNC+ovNC	shBRD1	shBRD1+ovSREBF1
repeat1	0.628792698	0.398484528	1.152865751
repeat2	0.543881525	0.202101211	0.860803828
repeat3	0.494948896	0.32433756	1.044524759
mean	0.555874373	0.308307766	1.019398113
normalization(/shNC)	0.999999951	0.554635663	1.833864112
SD	0.055295643	0.080970437	0.120550274

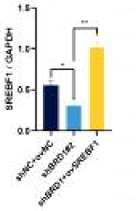

SCD1	shNC+ovNC	shBRD1	shBRD1+ovSREBF1
repeat1	1.015877879	0.421204294	0.633492936
repeat2	0.95377612	0.266530021	0.486314807
repeat3	1.15904561	0.385141596	0.790274497
mean	1.04289987	0.357625304	0.63669408
normalization(/shNC)	0.999999971	0.342914314	0.610503539
SD	0.08595166	0.066075172	0.124111667

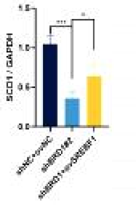

Figure 5A

Hep3B

H3K9ac	Blank	shNC	shBRD1#1	shBRD1#2
repeat1	0.849122881	1.037689	0.648591446	0.8649336
repeat2	0.770126603	0.751678	0.674461686	0.9574054
repeat3	1.084400765	0.792817	0.625536389	0.949008
mean	0.90121675	0.860728	0.64952984	0.9237823
normalization(/shNC)	1.047040157	1	0.75462848	1.073257
SD	0.133485057	0.126253	0.019984688	0.0417533

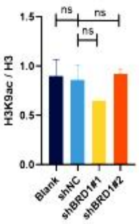

H3K14ac	Blank	shNC	shBRD1#1	shBRD1#2
repeat1	1.083630489	1.198362	0.763755618	0.5720705
repeat2	0.953525172	0.94204	0.691501214	0.5386682
repeat3	0.960290601	1.161133	0.48731208	0.762734
mean	0.999148754	1.100512	0.647522971	0.6244909
normalization(/shNC)	0.907894465	1	0.58838338	0.5674549
SD	0.059801424	0.113082	0.117063578	0.0986992

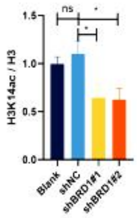

Supplement: Supplementary file 2 — Original data [file 41419_2025_7404_MOESM2_ESM.pdf]
